# Outdoor air pollution, road traffic noise, and allostatic load in children aged 6–11 years: evidence from six European cohorts

**DOI:** 10.1007/s10654-025-01227-8

**Published:** 2025-05-14

**Authors:** Yuchan Mou, Michelle Sofia Wilhelmina Kusters, Oliver Robinson, Lea Maitre, Rosemary R. C. McEachan, Wen Lun Yuan, Norun Hjertager Krog, Sandra Andrušaitytė, Mariona Bustamante, Montserrat de Castro Pascual, Audrius Dedele, John Wright, Regina Grazuleviciene, Gunn Marit Aasvang, Johanna Lepeule, Mark Nieuwenhuijsen, Henning Tiemeier, Martine Vrijheid, Errol M. Thomson, Mònica Guxens

**Affiliations:** 1 ISGlobal, Barcelona, Spain; 2 Department of Child and Adolescent Psychiatry/Psychology, Erasmus MC, University Medical Centre, Rotterdam, The Netherlands; 3 Department of Epidemiology, Erasmus MC, University Medical Center, Rotterdam, The Netherlands; 4 Universitat Pompeu Fabra, Barcelona, Spain; 5 Spanish Consortium for Research on Epidemiology and Public Health (CIBERESP), Instituto de Salud Carlos III, Madrid, Spain; 6 Μedical Research Council Centre for Environment and Health, Imperial College London, London, UK; 7 Mohn Centre for Children’s Health and Well-Being, School of Public Health, Imperial College London, London, UK; 8 Bradford Institute for Health Research, Bradford Teaching Hospitals NHS Foundation Trust, Bradford, UK; 9 Inserm U 1209, CNRS UMR 5309, Team of Environmental Epidemiology Applied to Development and Respiratory Health, Institute for Advanced Biosciences, University Grenoble Alpes, Grenoble, France; 10 Division of Climate and Environmental Health, Department of Air Quality and Noise, Norwegian Institute of Public Health, Oslo, Norway; 11 Department of Environmental Sciences, Vytautas Magnus University, Kaunas, Lithuania; 12 Inserm, INRAE, Centre for Research in Epidemiology and StatisticS (CRESS), Université Paris Cité and Université Sorbonne Paris Nord, 75004 Paris, France; 13 Department of Social and Behavioral Sciences, Harvard TH Chan School of Public Health, Boston, MA USA; 14 Environmental Health Science and Research Bureau, Healthy Environments and Consumer Safety Branch, Health Canada, Ottawa, Canada; 15 Department of Biochemistry, Microbiology and Immunology, Faculty of Medicine, University of Ottawa, Ottawa, Canada; 16 ICREA, Barcelona, Spain

**Keywords:** Allostasis, Air pollution, Particulate matter, Noise, Transportation, Environmental exposure, Children

## Abstract

Emerging evidence suggests that exposure to air pollutants and road traffic noise triggers stress responses, which mediate physiological responses in multiple organs and tissues. However, epidemiological study in children is sparse. We aimed to evaluate whether outdoor air pollution and road traffic noise are associated with physiological response measured by allostatic load in children. We studied 919 children aged 6–11 years from the HELIX (Human Early Life Exposome) cohort in 6 European countries with 19 biomarkers assessed in four physiological systems—cardiovascular, metabolic, immune/inflammation, and neuroendocrine systems. We then calculated both count-based and continuous scores for each physiological system and generated allostatic load scores (range 0–19). Exposure to air pollution (NO_2_, PM_2.5_, PM_10_, PM_2.5_absorbance) and road traffic noise (L_DEN_) based on participants’ home, school, and commuting route addresses were estimated for the year prior to outcome assessment. Higher exposure to all air pollutants was associated with a higher allostatic load, although only the association of PM_10_ survived correction for multiple testing (for count-based allostatic load score: *RR* = 1.27, 95%CI: 1.08, 1.48; for continuous allostatic load score: *β* = 0.56, 95% CI: 0.27–0.84, per each 10 µg/m^3^ increase in PM_10_). Examining physiological systems separately, higher exposure to air pollution was mainly associated with higher allostatic load in the immune/inflammatory and metabolic systems. No associations between road traffic noise and allostatic load were observed. Our findings suggest that air pollution act as a chronic stressor in manifesting multi-systemic physiological dysregulation in childhood, which may be a precursor of air pollution-related diseases.

**Supplementary Information:**

The online version contains supplementary material available at 10.1007/s10654-025-01227-8.

## Introduction

Air pollution is one of the greatest environmental risks to health and presented a great burden of disease globally over the past two decades [1]. Road traffic noise, the second most important driver of environmental burden of disease in Europe, has also been associated with increased risk of cardiometabolic diseases, cognitive impairment, and sleep disturbance [2]. Recent evidence suggests that effects on the stress response system may serve as a mechanism through which air pollution and noise lead to adverse health outcomes [3–5]. Experimental studies have demonstrated that exposure to air pollutants and road traffic noise elicits stress responses, including the activation of the hypothalamic–pituitary–adrenal (HPA) axis and the release of stress hormones, which then mediate physiological responses in multiple organ and tissues [3, 6]. Furthermore, chronic activation or dysregulation of the HPA axis has been associated with a wide variety of chronic diseases, including cardiovascular, pulmonary, metabolic, and neurological health outcomes [7].

The wear and tear on the body as it responds to stressors has been conceptualized as allostatic load [8], which involves the dysregulation of multiple physiological systems. Efforts to operationalize the concept of allostatic load often involve the use of composite indices that incorporate multisystemic biological measures sensitive to dysregulated physiological processes that can arise from chronic or cumulative stress. Accumulating evidence has demonstrated an association of allostatic load with all-cause mortality [9] and mental and cardiometabolic diseases in the adult population [10, 11]. Because they enable the monitoring of subclinical effects on multiple biological pathways, allostatic load constructs present a means to examine more fully how chronic exposure to environmental stressors may adversely impact health in ways that differ within a population according to individual sensitivity [12]. Indeed, on the basis of experimental evidence of pollutant-induced HPA axis activation, and the considerable overlap between stress-related diseases and those associated with air pollution, chronic activation and dysfunction of stress responses leading to increased allostatic load has been proposed as a common pathway contributing to adverse health outcomes associated with air pollution and other stressors [3, 13]. However, there is a paucity of epidemiological research examining the association of air pollution and road traffic noise with allostatic load, and studies of allostatic load in children are scarce.

In this study, we aimed to investigate associations of air pollution and road traffic noise with allostatic load in children aged 6–11 years in a multi-center longitudinal population-based cohort in Europe.

## Method

### Study design and population

This study used data from the Human Early Life Exposome (HELIX) cohort [14, 15], established across six longitudinal population-based birth cohorts: Born in Bradford (BiB), United Kingdom [16]; Étude des Déterminants pré et postnatals du développement et de la santé de l’Enfant (EDEN), France [17]; INfancia y Medio Ambiente (INMA), Spain [18]; Kaunas cohort (KANC), Lithuania [19]; Norwegian Mother, Father and Child Cohort (MoBa) [20]; and Rhea Mother Child Cohort (RHEA), Greece [21]. Mother–child pairs (N = 1,301) participated in a harmonized follow-up examination between December 2013 and February 2016, when children were 6–11 years old, as fully described elsewhere [15]. Eligibility criteria for inclusion were: (a) age 6–11 years at the time of the visit, with a preference for ages 7–9 years; (b) sufficient stored pregnancy blood and urine samples available for analysis of prenatal exposure biomarkers; (c) complete address history available from first to last follow-up point; (d) no serious health problems that may affect the clinical testing or impact the volunteer’s safety (e.g., acute respiratory infection); and (e) available data on important covariates (e.g., socioeconomic factors). Each cohort selected participants from the eligible pool in the entire cohort and invited them to participate in this subcohort until the required number of participants was reached. In total, 1,301 mother–child pairs were included in the HELIX subcohort. In our study, we included 919 children (around 150 from each cohort) who had information on all biomarkers included in the construction of allostatic load score and at least one exposure available (**eFig. 1**).

### Outdoor air pollutants

We estimated outdoor air pollutants the year before the allostatic load assessment. Nitrogen dioxide (NO_2_), particulate matter of less than 2.5 µm (PM_2.5_) and of less than 10 µm (PM_10_), and absorbance of PM_2.5_ filters (PM_2.5_absorbance) were assessed using land use regression (LUR) based on the addresses, temporally corrected by measurements from local background monitoring stations and averaged over the year of interest. For most cohorts and pollutants, we used site-specific LUR models developed within the European Study of Cohorts for Air Pollution Effects (ESCAPE) project [22, 23] (**eTable 1**). However, in EDEN, PM_2.5_ was estimated using the ELAPSE model [24]. And in BiB, PM_2.5_ and PM_10_ were estimated based on the ESCAPE LUR model developed in London/Oxford (UK) and adjusted for background PM levels from monitoring stations in Bradford [25]. EDEN and RHEA cohorts had no data for PM2.5 absorbance, nor did EDEN for PM10. Back-extrapolation based on other available pollutants was used when daily data on a pollutant was not available (**eTable 2**). Exposure was calculated as a weighted average over one year before the date of the follow-up examination for addresses of home, school, commuting routes, and other places gathered using an open-source Geographic Information System software aided by Google Maps [26].

### Road traffic noise

We estimated road traffic noise levels at home and school addresses for one year before the allostatic load assessment. Road traffic noise levels were estimated using L_den_, which is the annual average sound pressure level of a 24 h period: day, evening, and night, with a 5 decibel (dB) penalty for evening noise (19:00–23:00), and 10 dB penalty added to nighttime noise (23:00–07:00). L_den_ was derived from noise maps produced in each local municipality under the European Environmental Noise Directive (EC Directive 2002/49/EC (EUR-Lex)). The values were categorized into four categories (< 55 dB; 55–59.9 dB; 60–64.9 dB; $\ge$ 65 dB). EDEN had a large proportion of missing noise values (> 70%) and was excluded in the analyses.

### Biomarkers of allostatic load

Based on the literature on allostatic load theory, biological relevance to chronic stress in children [27], and data availability, we selected 19 biomarkers to create composite scores representing the allostatic load status of children. We grouped biomarkers into four physiological systems based on the allostatic load framework [28], namely cardiovascular, metabolic, immune/inflammatory, and neuroendocrine. Cardiovascular biomarkers included systolic blood pressure, diastolic blood pressure, and pulse rate. Metabolic biomarkers included body mass index (BMI), waist circumference, skinfold thickness, fat mass percentage, and plasma or serum levels of high-density lipoprotein cholesterol (HDL), non-HDL, triglycerides, leptin, and adiponectin. Immune/inflammatory markers included plasma levels of C-reactive protein (CRP), interleukin (IL)-1β, IL-6, IL-8, IL-10 and tumor necrosis factor (TNF)-α. The neuroendocrine biomarker was total cortisol production derived as the sum of cortisol level and related metabolites measured in urine samples. The detailed methods for measuring these biomarkers are outlined in the **Supplementary Methods**.

### Allostatic load scores

We defined two sets of sex-specific allostatic load scores as the composite measures of the 19 biomarkers, namely count-based allostatic load score and continuous allostatic load score. For the count-based allostatic load score, the highest quartile of each biomarker in the sex-stratified population distribution was considered ‘high risk’ of physiological dysregulation and received a score of 1, except for HDL, adiponectin, and IL-10, where the lowest quartile was considered ‘high risk’; and for total cortisol production, both lowest and highest 12.5th percentiles were labelled as ‘high risk’ [29]. The count-based allostatic load score was computed as a sum of the 19 individual 0/1 biomarker-risk scores. For the continuous allostatic load score, sex-stratified population distributions of biomarkers were rescaled to distributions between 0 and 1 (i.e., min–max normalization). For HDL, adiponectin, and IL-10, an individual’s biomarker risk score was computed by first rescaling their measurement to the interval [0,1] and then subtracting this rescaled value from 1. A risk score for total cortisol production was computed by first rescaling an individual’s derived cortisol sum to the interval [− 1,1] and then taking the absolute value of the rescaled score. The continuous allostatic load score was computed by taking the sum of continuous risk-scores across the collection of 19 biomarkers. Higher scores indicated higher risk. The cutoffs, medians, and interquartile ranges for each biomarker across all cohorts are provided in Table 1, and separately for each cohort in **eTable 3**. We calculated allostatic load scores for each physiological system and overall. Theoretical ranges of each score are determined by the number of biomarkers included (e.g. from 0 to 19 for the overall score).

**Table 1:** Percentiles of biomarkers of allostatic load grouped in four physiological systems

Biomarkers	Distributional cut-off points Median, percentiles [25th, 75th]		Continuous score^1^ Median, percentiles [25th, 75th]	
	Girls	Boys	Girls	Boys
*Cardiovascular system*				
Systolic blood pressure z score	− 0.05 (− 0.63, 0.57)	− 0.12 (− 0.74, 0.57)	0.37 (0.28, 0.46)	0.28 (0.20, 0.36)
Diastolic blood pressure z score	− 0.08 (− 0.57, 0.43)	− 0.19 (− 0.56, 0.27)	0.30 (0.22, 0.38)	0.22 (0.17, 0.29)
Pulse rate z score	0.04 (− 0.66, 0.66)	0.00 (− 0.63, 0.62)	0.39 (0.29, 0.48)	0.56 (0.46, 0.65)
*Metabolic system*				
BMI z score	0.36 (− 0.24, 1.17)	0.23 (− 0.42, 1.23)	0.44 (0.35, 0.56)	0.46 (0.38, 0.58)
Waist circumference z score	− 0.16 (− 0.66, 0.42)	− 0.28 (− 0.66, 0.32)	0.33 (0.26, 0.42)	0.55 (0.51, 0.62)
Skinfold thickness (Triceps & Subscapular)	17.93 (14.37, 22.92)	13.80 (11.57, 18.20)	0.18 (0.12, 0.27)	0.10 (0.07, 0.18)
Fat mass, percentage of total weight (%)	24.42 (19.43, 29.52)	20.39 (15.83, 25.93)	0.51 (0.40, 0.63)	0.38 (0.28, 0.51)
HDL, mg/L	58.01 (50.27, 65.74)	58.01 (50.27, 69.61)	0.60 (0.50, 0.70)	0.64 (0.5, 0.73)
Non-HDL, mg/L	104.41 (88.94, 119.88)	100.54 (85.07, 112.14)	0.39 (0.29, 0.49)	0.39 (0.29, 0.46)
Triglycerides, mg/L	77.06 (61.11, 103.63)	73.51 (56.68, 98.31)	0.20 (0.14, 0.30)	0.12 (0.07, 0.19)
Leptin, pg/ml	10.46 (9.73, 11.23)	9.65 (8.70, 10.55)	0.61 (0.49, 0.74)	0.56 (0.43, 0.69)
Adiponectin, pg/ml	24.58 (24.22, 24.86)	24.56 (24.18, 24.8)	0.21 (0.16, 0.28)	0.31 (0.24, 0.44)
*Immune/Inflammatory system*				
CRP, pg/ml	19.41 (18.14, 20.70)	18.79 (17.52, 20.21)	0.54 (0.39, 0.70)	0.52 (0.38, 0.67)
IL-1B, pg/ml	3.70 (3.28, 4.32)	3.16 (2.78, 3.74)	0.37 (0.28, 0.51)	0.27 (0.19, 0.39)
IL-6, pg/ml	3.56 (3.22, 4.10)	3.19 (2.87, 3.61)	0.42 (0.33, 0.56)	0.30 (0.23, 0.4)
IL-8, pg/ml	6.50 (6.35, 6.66)	6.46 (6.31, 6.61)	0.22 (0.18, 0.26)	0.19 (0.14, 0.23)
IL-10, pg/ml	2.37 (1.55, 3.39)	2.27 (1.55, 3.29)	0.71 (0.60, 0.80)	0.70 (0.6, 0.77)
TNF-a, pg/ml	4.93 (4.75, 5.09)	4.92 (4.75, 5.04)	0.18 (0.14, 0.21)	0.19 (0.16, 0.22)
*Neuroendocrine system*				
Cortisol production, µg/µmol creatinine, log10 transformed, median, [12.5th, 85.5th]	− 0.54 (− 0.90, − 0.13)	− 0.57 (− 0.93, − 0.11)	0.34 (0.09, 0.63)	0.29 (0.09, 0.57)

Abbreviations: HDL, high-density lipoprotein cholesterol; CRP, C-reactive protein; IL, interleukin; TNF, Tumor necrosis factor

^1^For the continuous allostatic load score, sex-stratified population distributions of biomarkers were rescaled to distributions between 0 and 1

### Covariates

Several covariates were considered as potential confounders based on previous literature and a directed acyclic graph (**eFig. 2**). Information on the covariates was collected during pregnancy and in the 6–11 years follow-up examination as follows: maternal and paternal education (low (primary school), middle (secondary school), high (university degree or higher)), parental country of origin (neither native, one native, both native to the country of the cohort), maternal marital and habitation status (living with the father, other situation), family’s economic capital measured with the Family Affluence Scale score (low (≤ 2), middle (3–5), high (≥ 6) scores) [30], parity (nulliparous, primiparous, multiparous), maternal alcohol drinking during pregnancy (yes, no), maternal smoking during pregnancy (yes, no), child sex, child age at the time of the examination, child ancestry (‘European’, ‘non-European’), child “moderate-to-vigorous physical activity” variable defined as the amount of time spent doing physical activities with intensity > 3 metabolic equivalent tasks (METs), child's sedentary behavior defined as the amount of time spent on any waking behavior characterized by an energy expenditure < 1.5 METs while in a sitting or reclining posture [31], and child exposure to second-hand smoking (yes, no).

### Statistical analysis

We used negative binomial regression models to examine associations of each outdoor air pollutant and road traffic noise, separately, with count-based allostatic load score, and linear regression models for continuous allostatic load score. All models were adjusted for all variables listed in the covariate section and by cohort. In addition, to test if scores from certain physiological systems were driving associations, we performed secondary analyses of the associations of air pollutants and scores for each physiological system. Model assumptions (i.e., Poisson response or normality of residuals, depending on the model, as well as linearity between exposure and outcome, and homoscedasticity) were checked and fulfilled.

To reduce potential selection bias due to missing values on covariates, we applied multiple imputation by generating 25 independent datasets with 50 iterations in each cohort separately using the ‘MICE’ package in R. We corrected for multiple testing by determining the eigenvalues to identify the effective number of tests (‘poolr’ package and ‘meff’ function in R), following the Galwey’s approach [32]. The effective number of tests was five for the main analyses (based on six exposures and two outcomes), making the new statistical significance level 0.05/5 = 0.01.

We performed several sensitivity analyses. First, we presented results from the minimally adjusted models accounting for cohort, child sex, age and ancestry. Second, we tested whether the associations were robust when considering only high cortisol values as ‘high risk’. Third, we ran the analyses in each cohort independently and performed random effects meta-analyses of each association. Fourth, we performed analyses of the relationship of exposure to air pollution at home address, school address, and commuting route separately with allostatic load, and of exposure to noise at home and school address separately with allostatic load. Fifth, since EDEN did not have information on PM_2.5_ absorbance and PM_10_ and RHEA did not have information on PM_2.5_ absorbance, we reran the analysis of NO_2_ and PM_2.5_ excluding EDEN, of NO_2_, PM_2.5_ and PM_10_ excluding RHEA, and of NO_2_, PM_2.5_ and PM_10_ excluding both EDEN and RHEA to evaluate the influence of the included cohorts in the results. All analysis were performed in R version 4.2.3 (R Foundation for Statistical Computing, Vienna, Austria).

## Results

The majority of parents were native to the country of the cohort **(**Table 2**)**. Half of the parents were highly educated, and the family had high family affluence. The characteristics of study population in each cohort are reported in **eTable 4**. The median of count-based allostatic load score for all children in the study was 4.0 (interquartile range 2.0, 7.0) and of continuous allostatic load score was 7.2 (6.5, 8.2). The two overall allostatic load scores were highly correlated (r = 0.89) and were both strongly correlated with their corresponding metabolic and immune/inflammatory physiological system scores (all r > 0.80), moderately correlated with cardiovascular system (r~0.40), and weakly with neuroendocrine (r~0.15) (**eFig. 3**, **eFig. 4)**.

**Table 2:** Characteristics of study population (N = 919)

Characteristics	N (%), mean (SD) or median [25th, 75th]
*Cohorts*	
BiB, UK	123 (13.4%)
EDEN, France	123 (13.4%)
INMA, Spain	183 (19.9%)
KANC, Lithuania	177 (19.3%)
MoBa, Norway	192 (20.9%)
Rhea, Greece	121 (13.2%)
*Child*	
Age, years	8.1 [6.5, 8.9]
Sex (Girls)	406 (44.2%)
Ancestry (European)	840 (91.4%)
Moderate-to-vigorous physical activity, mins/day	42.9 [17.1, 77.1]
Sedentary time, mins/day	212.1 [154.3, 291.4]
Exposure to second-hand smoking (Yes)	315 (34.3%)
*Mothers*	
Age, years	30.9 (4.8)
Smoking during pregnancy (Yes)	132 (14.4%)
Alcohol drinking during pregnancy (Yes)	290 (31.6%)
Parity	
Nulliparous	401 (43.6%)
Primiparous	346 (37.6%)
Multiparous	173 (18.8%)
*Parents*	
Marital and habitation status	
Living with the father	868 (94.5%)
Other situation	50 (5.5%)
Smoking status of parents	
Neither	578 (62.9%)
One	240 (26.1%)
Both	101 (11.0%)
*Socioeconomic status*	
Maternal educational level	
Low	115 (12.5%)
Middle	322 (35.0%)
High	482 (52.5%)
Paternal educational level	
Low	162 (17.6%)
Middle	362 (39.4%)
High	395 (43.0%)
Family Affluence Scale	
Low	85 (9.3%)
Middle	363 (39.5%)
High	471 (51.3%)
Parental national origin	
None or one native	136 (14.8%)
Both native	783 (85.2%)
*Allostatic load*	
Count-based allostatic load score	4.0 [2.0, 7.0]
Continuous allostatic load score	7.2 [6.5, 8.2]

Abbreviations: SD, standard deviation. Missing data of covariates were imputed with multiple imputation (25 imputations)

Levels of exposure to outdoor air pollutants and road traffic noise in each cohort are presented in Table 3. The correlations of levels of exposure to outdoor air pollutants and road traffic noise with biomarkers included in allostatic load scores ranged from -0.12 to 0.36 (**eFig. 5**). For all air pollutants, total exposure, exposure at home address and at school address were highly correlated (r > 0.80), while their associations with exposure on commuting routes were less strong, except for PM_2.5_ and PM_10_ (r > 0.75) (**eFig. 6)**. Exposure to road traffic noise at home address and school address were moderately correlated (r = 0.62).

**Table 3:** Levels of exposure to outdoor air pollutants and road traffic noise and allostatic load scores in each cohort

	BiB, UK	EDEN, France	INMA, Spain	KANC, Lithuania	MoBa, Norway	Rhea, Greece
Number of participants	123	123	183	177	192	121
Outdoor air pollutants, median [25th, 75th]						
NO_2_, µg/m^3^	31.5 (29.6, 33.9)	10.2 (9.1, 11.8)	35.9 (27.8, 40.5)	14.5 (13.3, 15.8)	26.4 (24, 30)	10 (8.7, 12)
PM_2.5_, µg/m^3^	14.5 (13.9, 15)	13.9 (12.1, 14.3)	13.5 (12.9, 14.1)	18.5 (17.8, 19.2)	7.8 (7.1, 9.1)	13.0 (12.1, 14.9)
PM_2.5_ absorbance, 10⁻^5^m⁻^1^	1.2 (1.1, 1.4)	N/A	1.7 (1.5, 2.0)	1.4 (1.3, 1.5)	0.9 (0.8, 1.0)	N/A
PM_10_, µg/m^3^	23.2 (22.3, 24.6)	N/A	28.9 (26.9, 30.7)	31.2 (29.9, 32.4)	12.5 (12.1, 12.7)	33.8 (31.8, 38.0)
Road traffic noise, n (%)						
Home						
< 55 dB	56.2	N/A	11.4	74.7	66.5	0.0
55–59.9 dB	28.9	N/A	21.1	18.2	17.8	6.1
60–64.9 dB	10.7	N/A	31.9	5.3	10.5	74.5
$\ge$ 65 dB	4.1	N/A	35.5	1.8	5.2	19.4
School						
< 55 dB	67.8	N/A	6.0	87.0	92.7	0.0
55–59.9 dB	23.1	N/A	24.7	11.8	5.2	4.1
60–64.9 dB	6.6	N/A	41.6	1.2	2.1	79.4
$\ge$ 65 dB	2.5	N/A	27.7	0.0	0.0	16.5

Abbreviations: NO2, nitrogen dioxide; PM, particulate matter; dB, decibel. Data not available was labelled as N/A in the table

**Fig. 1:**
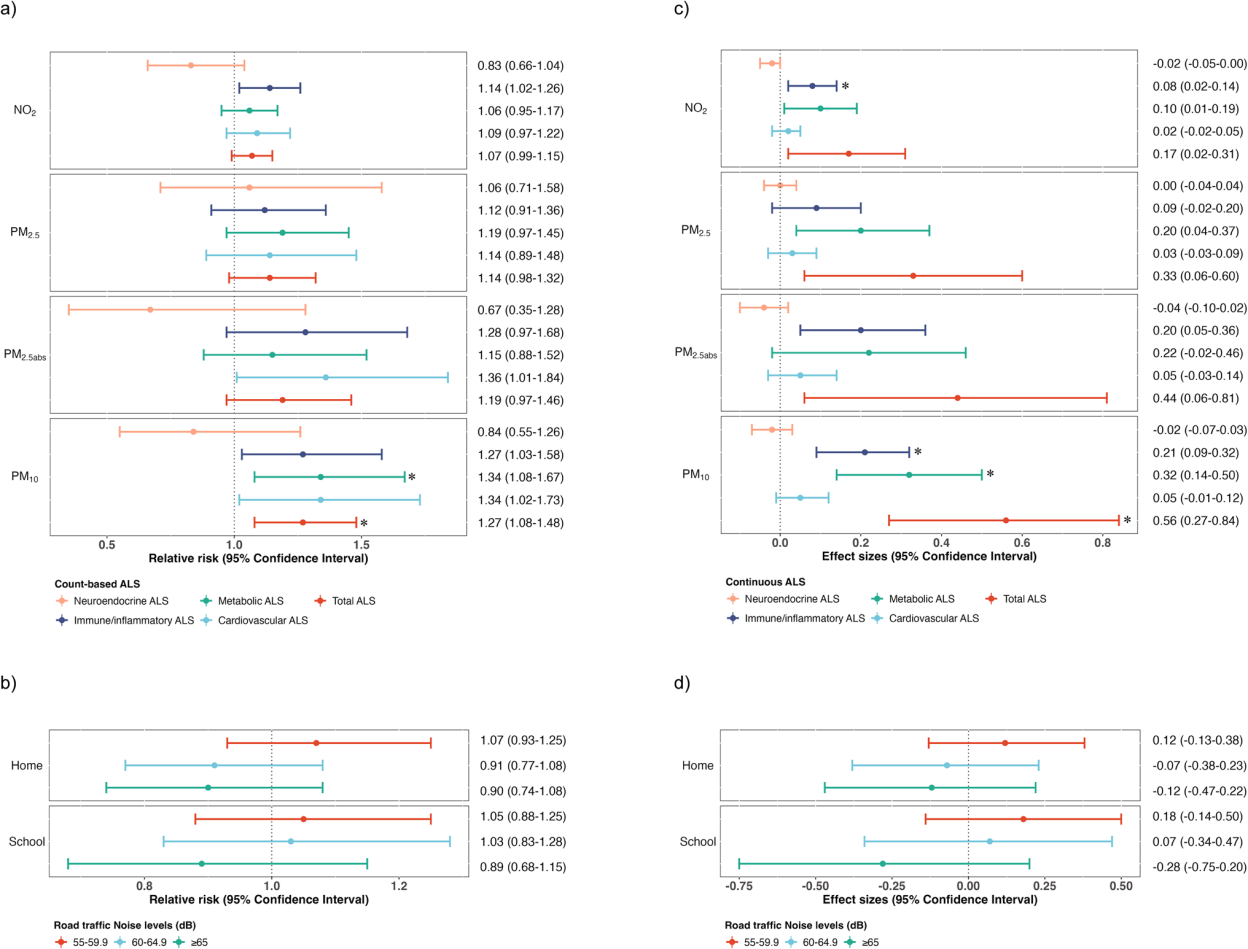
The associations of exposure to outdoor air pollutants and traffic noise with allostatic load. Abbreviations: ALS, allostatic load score; dB, decibel. Associations of outdoor air pollutants with (**a**) count-based allostatic load score, (**c**) continuous allostatic load score, and their associated allostatic load score per physiological systems; the associations of traffic noise levels with (**b**) count-based allostatic load score, (**d**) continuous allostatic load score. The models were adjusted for cohort, child sex, age, ancestry, physical activity, sedentary behaviors, exposure to second-hand smoking at the 6-11 years follow-up, the family’s economic capital, maternal and paternal education, parental country of origin, maternal marital status, parity, alcohol drinking during pregnancy, and active smoking during pregnancy. * denotes statistical significant after multiple testing correction (*p*-value 0.01)

Higher exposure to PM_10_ was associated with higher risk of allostatic load, and the association remained after correction for multiple testing (e.g., 27% increase (95%CI: 1.08, 1.48) in the risk of allostatic load score for every 10 µg/m^3^ increase in PM_10_) (Fig. 1a). We observed no associations between NO_2_, PM_2.5_, PM_2.5_ absorbance, road traffic noise** (**Fig. 1b), and count-based allostatic load score. Exposure to higher levels of each of the air pollutants was associated with higher continuous allostatic load, although only the association with PM_10_ survived multiple testing correction (e.g., 0.56 increase (95% CI: 0.27, 0.84) in the mean allostatic load score for every 10 µg/m^3^ increase in PM_10_) (Fig. 1c). No association was observed between road traffic noise and continuous allostatic load score (Fig. 1d**)**. When focusing on the allostatic load score of each physiological system, we found that higher exposure to PM_10_ was associated with higher allostatic load in the immune/inflammatory, and metabolic systems, and higher exposure to NO_2_ was associated with higher allostatic load in the immune/inflammatory system (Fig. 1a and c). No association was observed between road traffic noise and the allostatic load score of each physiological system (**eTables 5 and 6**).

The results from the minimally adjusted models (accounting for cohort, child sex, age, and ancestry) were consistent with the main results (**eTables 7 and 8**). We observed similar results when considering only high values of cortisol production as ‘high risk’ in allostatic load scores, with slightly larger effect estimates (**eTables 9 and 10**). We found similar results in the meta-analysis, with slight to moderate heterogeneity between cohorts (**eFig. 7**), suggesting that the effects were generally consistent across cohorts, and the main results were not likely to be driven by specific cohort. Associations of PM10 exposure were observed mainly for analyses using home and school addresses, but not for the commuting routes (**eFig. 8**). The effect estimates from the analysis excluding EDEN and RHEA were similar to the main results (**eFig. 9**).

## Discussion

The findings of this study suggest that higher exposure to PM_10_ was associated with an increase in allostatic load in children. This association was robust regardless of the approach used to operationalize allostatic load and adjusting for socioeconomic, lifestyle, and environmental factors. In addition, the results from the minimally adjusted models remained similar to the main results, supporting the robustness of the overall findings. Similar results were found in the meta-analysis combining cohort-specific effect estimates. Further analysis on testing the association by four physiological systems separately indicated that the association was mainly in the immune/inflammatory and metabolic systems. The observed associations were explained mainly by exposure to air pollutants at home and school of the children. We did not observe an association between exposure to road traffic noise and allostatic load.

To our knowledge, this is the first epidemiological study to describe associations between air pollution and allostatic load in children. Two prior observational studies investigated the associations of outdoor air pollution with allostatic load in teenagers and adults. One multicenter registry-based study of 2338 adolescents with type 1 diabetes in the US reported a positive association between annual average PM_2.5_ and allostatic load score [33]. Another large cross-sectional study of around 90,000 adults aged 30–79 years in a multicenter cohort in China found that long-term exposure to outdoor PM_2.5_ and PM_10_ was associated with an higher risk of allostatic overload [34]. Although there are differences in study population, sample size, exposure assessment, and operationalization of allostatic load, the direction of the effect of long-term air pollutant exposure in these two studies is consistent with what we observed in children.

Although few studies have examined the relationship between exposure to air pollution and biomarkers of allostatic load, the toxicological literature supports the biological plausibility of stress-based mechanisms underlying systemic health effects of air pollutants. Prior experimental studies have established that inhaling air pollutants activates the HPA axis and induces the release of glucocorticoid stress hormones, contributing to systemic metabolic and inflammatory effects and to altered transcriptional profiles across multiple tissues and organs [35–37]. Blocking glucocorticoid synthesis negated many metabolic and inflammatory responses to pollutant exposure [36], confirming that stress hormones mediate such effects. Panel studies have further verified that air pollutants [38–40] increased blood levels of glucocorticoid stress hormones. Our results, using allostatic load constructs consisting of multiple biomarkers as a system-wide chronic stress indicator, support the hypothesis that exposure to ambient pollutants induce stress responses that contribute to physiological dysfunction in childhood, which in turn may increase risk of disease at later ages. This is consistent with recent work that suggests perturbation of individual physiological and biochemical measures in children in relation to air pollution levels. For instance, a recent meta-analysis concluded that long-term exposures to air pollution may elevate systolic and diastolic blood pressure among children and adolescents [41, 42]. Epidemiological studies have found associations between air pollution and various metabolic (e.g., BMI, blood lipids) [43–46], and inflammatory (e.g., CRP, IL-10, IL-6, TNF) [47, 48] biomarkers in children. The present findings add to this growing body of evidence supporting the notion that environmental exposures may shape trajectories of health and disease from an early age.

While some previous studies have suggested associations of chronic noise exposure in childhood [42, 49] with blood pressure and stress hormone levels in children—although with less consistent results—we did not observe such associations in this study. Possible explanations for this lack of finding include other features of noise exposure, such as the type (e.g., aircraft noise) [50], the frequency of noise [51], and noise fluctuation characteristics [52] that may be important in evaluating the health impact of noise as seen in other studies, information that we lack.

Our study has several strengths. First, we operationalized allostatic load encompassing a broad range of biomarkers and applying an identification of ‘risk’ in physiological dysregulation in a sex-dependent manner. In addition, since there is a lack of consensus on the calculation of the allostatic load score, we explored two scoring strategies that yielded similar results. Second, we assessed children’s exposure at different locations, which captured a relatively complete actual outdoor exposure given their daily activity patterns. Third, the analyses were conducted on harmonized data obtained from six cohorts in Europe, enhancing the robustness of the findings across diverse populations.

Our results should be interpreted in the context of its limitations. First, we included urinary cortisol production as a biomarker of effects on the neuroendocrine system. The urinary sample in our study was collected at unified times before bedtime to minimize diurnal variation, and multiple cortisol metabolites were included. However, cortisol exhibits high within-individual variability and measurement in urine reflects cortisol output over a short-term period. Other observational studies measuring cortisol concentration using hair samples [53] have found associations between air pollutants with cortisol. Second, we estimated children’s exposure using modeled maps. Air pollution campaigns were performed some years before the allostatic load assessment and pollutants were extrapolated to the study period of interest. This was based on the assumption that air pollution levels remain spatially constant over time, as supported by some studies [54, 55]. Also, road traffic noise estimates were based on annual averages not capturing noise fluctuations or other noise events that have been shown to be physiologically more disruptive [56]. Third, we cannot rule out potential residual confounding due to the observational nature of the study. While we adjusted for various individual-level factors to account for socioeconomic status, we did not control for neighborhood-level variables limited by data availability, such as neighborhood deprivation index, which has been independently linked to air pollution and traffic noise [57]. Last, this study population was drawn from the subcohort selected from the HELIX study, resulting in a population with a higher socioeconomic status [15]. Therefore, future studies are needed to confirm the generalizability of our findings in different contexts.

## Conclusion

This study found that associations between outdoor air pollution and allostatic load can be detected in mid-childhood. Our findings suggest air pollutants act as chronic stressors in the manifestation of multi-systemic physiological dysregulation in childhood, which may be the precursor of air pollution-related diseases. As allostatic load is predictive of trajectories of health and disease, childhood exposures present a potential target for intervention to reduce future disease burden.

## Supplementary Information

Below is the link to the electronic supplementary material.

Supplementary file1 (DOCX 3666 KB)

## References

[1] Cohen AJ, Brauer M, Burnett R, et al. Estimates and 25-year trends of the global burden of disease attributable to ambient air pollution: an analysis of data from the global burden of diseases study 2015. Lancet. 2017;389(10082):1907–18. 10.1016/S0140-6736(17)30505-6. 28408086 10.1016/S0140-6736(17)30505-6 PMC5439030

[2] World Health Organization. Regional Office for E. Environmental noise guidelines for the European Region. Copenhagen: World Health Organization. Regional Office for Europe; 2018.

[3] Thomson EM. Air pollution, stress, and allostatic load: linking systemic and central nervous system impacts. J Alzheimers Dis. 2019;69:597–614. 10.3233/JAD-190015. 31127781 10.3233/JAD-190015 PMC6598002

[4] Snow SJ, Henriquez AR, Costa DL, Kodavanti UP. Neuroendocrine regulation of air pollution health effects: emerging insights. Toxicol Sci. 2018;164(1):9–20. 10.1093/toxsci/kfy129. 29846720 10.1093/toxsci/kfy129 PMC6659011

[5] Sørensen M, Pershagen G, Thacher JD, et al. Health position paper and redox perspectives - disease burden by transportation noise. Redox Biol. 2024;69: 102995. 10.1016/j.redox.2023.102995. 38142584 10.1016/j.redox.2023.102995 PMC10788624

[6] Recio A, Linares C, Banegas JR, Díaz J. Road traffic noise effects on cardiovascular, respiratory, and metabolic health: an integrative model of biological mechanisms. Environ Res. 2016;146:359–70. 10.1016/j.envres.2015.12.036. 26803214 10.1016/j.envres.2015.12.036

[7] Chrousos GP. Stress and disorders of the stress system. Nat Rev Endocrinol. 2009;5(7):374–81. 10.1038/nrendo.2009.106. 19488073 10.1038/nrendo.2009.106

[8] McEwen BS. Protective and damaging effects of stress mediators. N Engl J Med. 1998;338(3):171–9. 10.1056/NEJM199801153380307. 9428819 10.1056/NEJM199801153380307

[9] Guidi J, Lucente M, Sonino N, Fava GA. Allostatic load and its impact on health: a systematic review. Psychother Psychosom. 2021;90(1):11–27. 10.1159/000510696. 32799204 10.1159/000510696

[10] Juster R-P, McEwen BS, Lupien SJ. Allostatic load biomarkers of chronic stress and impact on health and cognition. Neurosci Biobehav Rev. 2010;35(1):2–16. 10.1016/j.neubiorev.2009.10.002. 19822172 10.1016/j.neubiorev.2009.10.002

[11] Seeman TE, Singer BH, Rowe JW, Horwitz RI, McEwen BS. Price of adaptation–allostatic load and its health consequences. MacArthur studies of successful aging. Arch Intern Med. 1997;157(19):2259–68. 9343003

[12] Thomson EM, Kalayci H, Walker M. Cumulative toll of exposure to stressors in Canadians: an allostatic load profile. Health Rep. 2019;30(6):14–21. 10.25318/82-003-x201900600002-eng. 31216048 10.25318/82-003-x201900600002-eng

[13] Thomson EM. Neurobehavioral and metabolic impacts of inhaled pollutants. Endocrine Disruptors. 2013;1(1): e27489. 10.4161/endo.27489.

[14] Vrijheid M, Slama R, Robinson O, et al. The human early-life exposome (HELIX): project rationale and design. Environ Health Perspect. 2014;122(6):535–44. 10.1289/ehp.1307204. 24610234 10.1289/ehp.1307204 PMC4048258

[15] Léa M, de Jeroen B, Maribel C, et al. Human early life exposome (HELIX) study: a European population-based exposome cohort. BMJ Open. 2018;8(9): e021311. 10.1136/bmjopen-2017-021311. 10.1136/bmjopen-2017-021311 PMC6144482 30206078

[16] McEachan RRC, Santorelli G, Watmuff A, et al. Cohort profile update: born in Bradford. Int J Epidem. 2024;53(2):dyae037. 10.1093/ije/dyae037. 10.1093/ije/dyae037 PMC11065350 38552669

[17] Heude B, Forhan A, Slama R, et al. Cohort Profile: The EDEN mother-child cohort on the prenatal and early postnatal determinants of child health and development. Int J Epidemiol. 2016;45(2):353–63. 10.1093/ije/dyv151. 26283636 10.1093/ije/dyv151

[18] Guxens M, Ballester F, Espada M, et al. Cohort Profile: the INMA–INfancia y Medio Ambiente–(Environment and Childhood) Project. Int J Epidemiol. 2012;41(4):930–40. 10.1093/ije/dyr054. 21471022 10.1093/ije/dyr054

[19] Grazuleviciene R, Danileviciute A, Nadisauskiene R, Vencloviene J. Maternal smoking, GSTM1 and GSTT1 polymorphism and susceptibility to adverse pregnancy outcomes. Int J Environ Res Public Health. 2009;6(3):1282–97. 10.3390/ijerph6031282. 19440446 10.3390/ijerph6031282 PMC2672398

[20] Magnus P, Birke C, Vejrup K, et al. Cohort profile update: the norwegian mother and child cohort study (MoBa). Int J Epidemiol. 2016;45(2):382–8. 10.1093/ije/dyw029. 27063603 10.1093/ije/dyw029

[21] Chatzi L, Leventakou V, Vafeiadi M, et al. Cohort profile: the mother-child cohort in crete, Greece (Rhea Study). Int J Epidemiol. 2017;46(5):1392–3. 10.1093/ije/dyx084. 29040580 10.1093/ije/dyx084

[22] Beelen R, Hoek G, Pebesma E, Vienneau D, De Hoogh K, Briggs DJ. Mapping of background air pollution at a fine spatial scale across the European Union. Sci Total Environ. 2009;407(6):1852–67. 19152957 10.1016/j.scitotenv.2008.11.048

[23] Beelen R, Hoek G, Vienneau D, et al. Development of NO2 and NOx land use regression models for estimating air pollution exposure in 36 study areas in Europe – The ESCAPE project. Atmos Environ. 2013;72:10–23. 10.1016/j.atmosenv.2013.02.037.

[24] de Hoogh K, Chen J, Gulliver J, et al. Spatial PM(2.5), NO(2), O(3) and BC models for Western Europe - evaluation of spatiotemporal stability. Environ Int. 2018;120:81–92. 10.1016/j.envint.2018.07.036. 30075373 10.1016/j.envint.2018.07.036

[25] Schembari A, de Hoogh K, Pedersen M, et al. Ambient air pollution and newborn size and adiposity at birth: differences by maternal ethnicity (the born in bradford study cohort). Environ Health Perspect. 2015;123(11):1208–15. 10.1289/ehp.1408675. 25978617 10.1289/ehp.1408675 PMC4629735

[26] Team QD. QGIS geographic information system. Open source geospatial foundation project.

[27] Condon EM. Chronic stress in children and adolescents: a review of biomarkers for use in pediatric research. Biol Res Nurs. 2018;20(5):473–96. 10.1177/1099800418779214. 29865855 10.1177/1099800418779214 PMC6346321

[28] McEwen BS. Protective and damaging effects of stress mediators: central role of the brain. Dialogues Clin Neurosci. 2006;8(4):367–81. 10.31887/DCNS.2006.8.4/bmcewen. 17290796 10.31887/DCNS.2006.8.4/bmcewen PMC3181832

[29] Wing SE, Bandoli G, Telesca D, Su JG, Ritz B. Chronic exposure to inhaled, traffic-related nitrogen dioxide and a blunted cortisol response in adolescents. Environ Res. 2018;163:201–7. 10.1016/j.envres.2018.01.011. 29454852 10.1016/j.envres.2018.01.011 PMC5878732

[30] Boyce W, Torsheim T, Currie C, Zambon A. The family affluence scale as a measure of national wealth: validation of an adolescent self-report measure. Soc Indic Res. 2006;78(3):473–87. 10.1007/s11205-005-1607-6.

[31] Tremblay MS, Aubert S, Barnes JD, et al. Sedentary behavior research network (sbrn) – terminology consensus project process and outcome. Int J Behav Nutr Phys Act. 2017;14(1):75. 10.1186/s12966-017-0525-8. 28599680 10.1186/s12966-017-0525-8 PMC5466781

[32] Galwey NW. A new measure of the effective number of tests, a practical tool for comparing families of non-independent significance tests. Genet Epidemiol. 2009;33(7):559–68. 10.1002/gepi.20408. 19217024 10.1002/gepi.20408

[33] Montresor-López JA, Reading SR, Yanosky JD, et al. The relationship between traffic-related air pollution exposures and allostatic load score among youth with type 1 diabetes in the SEARCH cohort. Environ Res. 2021;197: 111075. 10.1016/j.envres.2021.111075. 33798519 10.1016/j.envres.2021.111075 PMC8187288

[34] Xu H, Yang T, Guo B, et al. Increased allostatic load associated with ambient air pollution acting as a stressor: cross-sectional evidence from the China multi-ethnic cohort study. Sci Total Environ. 2022;831: 155658. 10.1016/j.scitotenv.2022.155658. 35523330 10.1016/j.scitotenv.2022.155658

[35] Thomson EM, Vladisavljevic D, Mohottalage S, Kumarathasan P, Vincent R. Mapping acute systemic effects of inhaled particulate matter and ozone: multiorgan gene expression and glucocorticoid activity. Toxicolog Sci. 2013;135(1):169–81. 10.1093/toxsci/kft137 PMC3748763 23805001

[36] Thomson EM, Pal S, Guenette J, et al. Ozone inhalation provokes glucocorticoid-dependent and-independent effects on inflammatory and metabolic pathways. Toxicol Sci. 2016;152(1):17–28. 27037194 10.1093/toxsci/kfw061 PMC12077420

[37] Thomson EM, Pilon S, Guénette J, Williams A, Holloway AC. Ozone modifies the metabolic and endocrine response to glucose: Reproduction of effects with the stress hormone corticosterone. Toxicol Appl Pharmacol. 2018;342:31–8. 29391239 10.1016/j.taap.2018.01.020

[38] Miller DB, Ghio AJ, Karoly ED, et al. Ozone exposure increases circulating stress hormones and lipid metabolites in humans. Am J Respir Crit Care Med. 2016;193(12):1382–91. 10.1164/rccm.201508-1599OC. 26745856 10.1164/rccm.201508-1599OC PMC5440058

[39] Li H, Cai J, Chen R, et al. Particulate matter exposure and stress hormone levels: a randomized, double-blind. Crossover Trial Air Purif Circul. 2017;136(7):618–27. 10.1161/circulationaha.116.026796. 10.1161/CIRCULATIONAHA.116.026796 28808144

[40] Thomson EM, Filiatreault A, Williams A, Rider CF, Carlsten C. Exposure to diesel exhaust and plasma cortisol response: a randomized double-blind crossover study. Environ Health Perspect. 2021;129(3):37701. 10.1289/ehp8923. 33769847 10.1289/EHP8923 PMC7997608

[41] Huang M, Chen J, Yang Y, Yuan H, Huang Z, Lu Y. Effects of ambient air pollution on blood pressure among children and adolescents: a systematic review and meta-analysis. J Am Heart Assoc. 2021;10(10): e017734. 10.1161/JAHA.120.017734. 33942625 10.1161/JAHA.120.017734 PMC8200690

[42] Warembourg C, Nieuwenhuijsen M, Ballester F, et al. Urban environment during early-life and blood pressure in young children. Environ Int. 2021;146: 106174. 10.1016/j.envint.2020.106174. 33099063 10.1016/j.envint.2020.106174

[43] Zhang Z, Dong B, Chen G, et al. Ambient air pollution and obesity in school-aged children and adolescents: a multicenter study in China. Sci Total Environ. 2021;771: 144583. 10.1016/j.scitotenv.2020.144583. 33524680 10.1016/j.scitotenv.2020.144583

[44] Wilding S, Ziauddeen N, Smith D, Roderick P, Chase D, Alwan NA. Are environmental area characteristics at birth associated with overweight and obesity in school-aged children? Findings from the SLOPE (Studying Lifecourse Obesity PrEdictors) population-based cohort in the south of England. BMC Med. 2020;18(1):43. 10.1186/s12916-020-01513-0. 32188454 10.1186/s12916-020-01513-0 PMC7081603

[45] Vrijheid M, Fossati S, Maitre L, et al. Early-life environmental exposures and childhood obesity: an exposome-wide approach. Environ Health Perspect. 2020;128(6):67009. 10.1289/ehp5975. 32579081 10.1289/EHP5975 PMC7313401

[46] Gui ZH, Yang BY, Zou ZY, et al. Exposure to ambient air pollution and blood lipids in children and adolescents: a national population based study in China. Environ Pollut. 2020;266(Pt 3): 115422. 10.1016/j.envpol.2020.115422. 32829032 10.1016/j.envpol.2020.115422

[47] Gruzieva O, Merid SK, Gref A, et al. Exposure to traffic-related air pollution and serum inflammatory cytokines in children. Environ Health Perspect. 2017;125(6): 067007. 10.1289/ehp460. 28669936 10.1289/EHP460 PMC5714301

[48] Puett RC, Yanosky JD, Mittleman MA, et al. Inflammation and acute traffic-related air pollution exposures among a cohort of youth with type 1 diabetes. Environ Int. 2019;132: 105064. 10.1016/j.envint.2019.105064. 31419765 10.1016/j.envint.2019.105064 PMC7717111

[49] Hohmann C, Grabenhenrich L, de Kluizenaar Y, et al. Health effects of chronic noise exposure in pregnancy and childhood: a systematic review initiated by ENRIECO. Int J Hyg Environ Health. 2013;216(3):217–29. 10.1016/j.ijheh.2012.06.001. 22854276 10.1016/j.ijheh.2012.06.001

[50] Thompson R, Smith RB, Bou Karim Y, et al. Noise pollution and human cognition: an updated systematic review and meta-analysis of recent evidence. Environ Int. 2022;158: 106905. 10.1016/j.envint.2021.106905. 34649047 10.1016/j.envint.2021.106905

[51] Baliatsas C, van Kamp I, van Poll R, Yzermans J. Health effects from low-frequency noise and infrasound in the general population: Is it time to listen? A systematic review of observational studies. Sci Total Environ. 2016;557–558:163–9. 10.1016/j.scitotenv.2016.03.065. 26994804 10.1016/j.scitotenv.2016.03.065

[52] Foraster M, Esnaola M, López-Vicente M, et al. Exposure to road traffic noise and cognitive development in schoolchildren in Barcelona, Spain: a population-based cohort study. PLoS Med. 2022;19(6): e1004001. 10.1371/journal.pmed.1004001. 35653430 10.1371/journal.pmed.1004001 PMC9162347

[53] Verheyen VJ, Remy S, Bijnens EM, et al. Long-term residential exposure to air pollution is associated with hair cortisol concentration and differential leucocyte count in Flemish adolescent boys. Environ Res. 2021;201: 111595. 10.1016/j.envres.2021.111595. 34186082 10.1016/j.envres.2021.111595

[54] Gulliver J, de Hoogh K, Hansell A, Vienneau D. Development and back-extrapolation of NO2 land use regression models for historic exposure assessment in Great Britain. Environ Sci Technol. 2013;47(14):7804–11. 10.1021/es4008849. 23763440 10.1021/es4008849

[55] Eeftens M, Beelen R, Fischer P, Brunekreef B, Meliefste K, Hoek G. Stability of measured and modelled spatial contrasts in NO(2) over time. Occup Environ Med. 2011;68(10):765–70. 10.1136/oem.2010.061135. 21285243 10.1136/oem.2010.061135

[56] Foraster M, Eze IC, Schaffner E, et al. Exposure to road, railway, and aircraft noise and arterial stiffness in the SAPALDIA study: annual average noise levels and temporal noise characteristics. Environ Health Perspect. 2017;125(9): 097004. 28934719 10.1289/EHP1136 PMC5915209

[57] Chi GC, Hajat A, Bird CE, et al. Individual and neighborhood socioeconomic status and the association between air pollution and cardiovascular disease. Environ Health Perspect. 2016;124(12):1840–7. 10.1289/ehp199. 27138533 10.1289/EHP199 PMC5132637

