# Supplementary material for: Outdoor air pollution, road traffic noise, and allostatic load in children aged 6–11 years: evidence from six European cohorts
Source: Eur J Epidemiol. 2025 May 14;40(5):537–48. doi: 10.1007/s10654-025-01227-8 (PMC12170719; doi:10.1007/s10654-025-01227-8)
Supplement: Supplementary file 1 — Supplementary file1 (DOCX 3666 KB) [file 10654_2025_1227_MOESM1_ESM.docx]

**Online Supplementary Material**

**Air pollution, road traffic noise, and allostatic load in children at age 6-11 years**

Yuchan Mou, Michelle Kusters, Oliver Robinson, Lea Maitre, Rosemary R C McEachan, Johanna Lepeule, Norun Hjertager Krog, Sandra Andrušaitytė, Mariona Bustamante, Montserrat de Castro Pascual, Audrius Dedele, John Wright, Wen Lun Yuan, Regina Grazuleviciene, Gunn Marit Aasvang, Mark Nieuwenhuijsen, Henning Tiemeier, Martine Vrijheid, Errol M. Thomson, Mònica Guxens

**Table of Contents**

Supplementary Methods. Measurement of biomarkers included in the operationalization of the allostatic load score

### eTable 1. Air pollution model used in each cohort

eTable 2. Availability of daily measurements for outdoor air pollutants from background monitoring stations, by cohort

eTable 3. Percentiles of biomarkers of allostatic load grouped in four physiological systems in each cohort

eTable 4. Characteristics of study population in each cohort

eTable 5. Adjusted relative risk of count-based allostatic load score of each physiological system associated with road traffic noise levels

eTable 6. Adjusted effect estimates of continuous allostatic load score of each physiological system associated with road traffic noise levels

eTable 7. Minimally adjusted relative risk of count-based allostatic load score associated with outdoor air pollutants and road traffic noise levels

eTable 8. Minimally adjusted effect estimates of continuous allostatic load score associated with outdoor air pollutants and road traffic noise levels

eTable 9. Adjusted relative risk of count-based allostatic load score associated with outdoor air pollutants and road traffic noise levels, when considering only high values of cortical production as ‘high risk’ in allostatic load scores

eTable 10. Adjusted effect estimates of continuous allostatic load score associated with outdoor air pollutants and road traffic noise levels, when considering only high values of cortical production as ‘high risk’ in allostatic load scores

eFig 1. Flow chart of the study population selection

eFig 2. Directed acyclic graph for confounder selection

eFig 3. Correlations of biomarkers included in allostatic load scores

eFig 4. Correlations of allostatic load scores

eFig 5. Correlations between levels of exposure to source-specific outdoor air pollutants and traffic noise and biomarkers included in allostatic load scores

eFig 6. Correlations of levels of exposure to source-specific outdoor air pollutants and traffic noise

eFig 7. Meta-analysis of associations between exposure to outdoor air pollutants, traffic noise and allostatic load stratified by cohort

eFig 8. The associations of exposure to outdoor air pollutants at home, school and commuting route addresses with allostatic load

eFig 9. The associations of exposure to outdoor air pollutants with allostatic load (excluding data from EDEN, RHEA separately and jointly)

### Supplementary Methods. Measurement of biomarkers included in the operationalization of the allostatic load score

Blood pressure was measured using an automated digital monitor (OMRON 705-CPII). After five minutes of rest, three consecutive measurements were taken in sitting position with one-minute intervals. We used the second and third measurements to reduce measurement error related to within person variability, and we derived z-scores of systolic and diastolic blood pressures using regression model for standardization by age, sex, and height. Pulse rate was standardized using regression by age and sex.

HDL and triglycerides levels were assessed with serum samples using homogeneous enzymatic colorimetric methods in the MODULAR ANALYTICS system (Roche Diagnostics) according to the manufacturer’s instructions. We calculated non-HDL by subtracting HDL from total cholesterol.

Leptin, adiponectin, CRP, IL-1β, IL-6, IL-8, IL-10, and TNF-α were measured with plasma samples. A set of proteins, including the abovementioned ones, were selected a priori based on the literature and on the Luminex kits commercially available from Life Technologies and Millipore. Three kits were selected for subsequent analyses: Cytokines 30-plex (Cat #. LHC6003M), Apoliprotein 5-plex (LHP0001M) and Adipokine 15-plex (LHC0017M). Plasma samples were analysed in the CRG/UPF Proteomics Unit with the xMAP and Luminex system and following the manufacturer’s protocol. The experimental design was designed to control for batch effects and confounding variables. For protein quantification, an 8-point calibration curve per plate was performed with protein standards provided in the Luminex kit and following the procedures described in the standard procedures described by the vendor. All samples were diluted ½ for the 30-plex kit, ¼ for the 15-plex kit and 1/2500 for the 5-plex kit. For each protein, the limit of detection (LOD) was determined and the lower and upper quantification limits (LOQ1 and LOQ2, respectively) were obtained from the calibration curves. The plate batch effect was corrected by subtracting for each individual and each protein the difference between the overall protein average minus the plate specific protein average. Finally, values above LOQ1 and below LOQ2 were imputed using a truncated normal distribution implemented in the truncdist R package [1]. The protein data was log transformed, imputed and normalized in the analysis.

Height and weight were measured using regularly calibrated instruments and converted to BMI age-and-sex–standardized *z*-scores (zBMI) using the international World Health Organization (WHO) reference curves [2]. Waist circumference was measured in duplicate to the nearest 0.1 cm in a standing position, at the high point of the iliac crest at the end of a gentle expiration, with the use of a measuring tap (Seca 201; Seca Coporation). Skinfold thickness was measured at two anatomic sites (subscapular and triceps) on the right side of the body in triplicate to the nearest 0.1 millimeter with a calibrated caliper and then we summed these two skinfolds as a score of subcutaneous fatness. To estimate body composition, bioelectric impedance analyses were performed with the Bodystat 1500 (Bodystat Ltd.) equipment after 5 minutes. The proportion of fat mass was calculated using published age- and race-specific equations validated for use in children [3].

Steroid profile, including information of levels of steroid hormone and metabolites, was determined with urine samples. The urine samples were collected at the last void before bedtime to reduce the effect of diurnal variation. To assess levels of cortisol and related metabolites, LC-MS/MS analysis was applied to night urine samples. The laboratory protocols for the analyses are described elsewhere [4]. Data below LOQ and not detected were replaced with half of the lower LOQ. Steroid hormones and metabolites were further normalized to account for variable concentration levels, with their value divided by corresponding sample creatinine levels. Total cortisol production was derived with the following formula: Ʃ (cortisol + 20α-dihydrocortisol + 20β-dihydrocortisol + 5β-dihydrocortisol + 5α-tetrahydrocortisol + 5β-tetrahydrocortisol + 6β-hydroxycortisol + 5α,20α-cortol +5α,20β-cortol + 5β,20α-cortol + 5β,20β-cortol), and was log transformed in the analysis.

**References**

1. Nadarajah S, Kotz S. The Exponentiated Type Distributions. Acta Applicandae Mathematica. 2006;92(2):97-111. doi:10.1007/s10440-006-9055-0

2. de Onis M, Onyango AW, Borghi E, Siyam A, Nishida C, Siekmann J. Development of a WHO growth reference for school-aged children and adolescents. Bull World Health Organ. 2007;85(9):660-7. doi:10.2471/blt.07.043497

3. Clasey JL, Bradley KD, Bradley JW, Long DE, Griffith JR. A New BIA Equation Estimating the Body Composition of Young Children. Obesity. 2011;19(9):1813-7. doi:https://doi.org/10.1038/oby.2011.158

4. Marcos J, Renau N, Casals G, Segura J, Ventura R, Pozo OJ. Investigation of endogenous corticosteroids profiles in human urine based on liquid chromatography tandem mass spectrometry. Anal Chim Acta. 2014;812:92-104. doi:10.1016/j.aca.2013.12.030

### eTable 1. Air pollution model used in each cohort

| Cohort | NO_2_ | PM_2.5_ | PM_2.5_ absorbance | PM_10_ |
| --- | --- | --- | --- | --- |
| BiB, UK | ESCAPE local LUR | ESCAPE London/Oxford LUR | ESCAPE local LUR | ESCAPE London/Oxford LUR |
| EDEN, France | ESCAPE local LUR | ELAPSE | N/A | N/A |
| INMA, Spain | ESCAPE local LUR | ESCAPE local LUR | ESCAPE local LUR | ESCAPE local LUR |
| KANC, Lithuania | ESCAPE local LUR | ESCAPE local LUR | ESCAPE local LUR | ESCAPE local LUR |
| MoBa, Norway | ESCAPE local LUR | ESCAPE local LUR | ESCAPE local LUR | ESCAPE local LUR |
| Rhea, Greece | ESCAPE local LUR | ESCAPE local LUR | N/A | ESCAPE local LUR |

Abbreviations: NO, nitrogen oxide; PM, particulate matter.

### eTable 2. Availability of daily measurements for outdoor air pollutants from background monitoring stations, by cohort

| Cohort | NO_2_ | PM_2.5_ | PM_2.5_ absorbance | PM_10_ |
| --- | --- | --- | --- | --- |
| BiB, UK | Daily values available | Back extrapolated (NO_2_) | Back extrapolated (NO_x_) | Back extrapolated (NO_2_) |
| EDEN, France | Daily values available | Back extrapolated (NO_2_) | N/A | N/A |
| INMA, Spain | Daily values available | Back extrapolated (NO_2_) | Back extrapolated (NO_x_) | Back extrapolated (NO_2_) |
| KANC, Lithuania | Daily values available | Back extrapolated (NO_2_) | Back extrapolated (NO_x_) | Daily values available |
| MoBa, Norway | Daily values available | Daily values available | Back extrapolated (NO_x_) | Daily values available |
| Rhea, Greece | Back extrapolated (PM_2.5_) | Back extrapolated (PM_10_) | N/A | Daily values available |

Abbreviations: NO, nitrogen oxide; PM, particulate matter. Data on daily background concentrations of air pollutants for temporal adjustment were obtained from routine background stations. Back-extrapolation based on other available pollutants was used when daily data on a pollutant was not available.

**eTable 3. Percentiles of biomarkers of allostatic load grouped in four physiological systems in each cohort**

| Biomarkers | Distributional cut-off points  Median, percentiles [25^th^, 75^th^] | | Continuous score  Median, percentiles [25^th^, 75^th^] | |
| --- | --- | --- | --- | --- |
|  | Girls | Boys | Girls | Boys |
| BiB, UK |  |  |  |  |
| *Cardiovascular system* |  |  |  |  |
| Systolic blood pressure z score | 0.37 (-0.18, 1.25) | 0.35 (-0.23, 1.06) | 0.43 (0.35, 0.57) | 0.34 (0.26, 0.42) |
| Diastolic blood pressure z score | 0.30 (-0.22, 1.02) | 0.2 (-0.22, 1.07) | 0.36 (0.27, 0.48) | 0.28 (0.22, 0.4) |
| Pulse rate z score | -0.06 (-1.05, 0.67) | -0.2 (-1, 0.38) | 0.38 (0.23, 0.48) | 0.53 (0.41, 0.62) |
| *Metabolic system* |  |  |  |  |
| BMI z score | 0.04 (-0.21, 0.78) | 0.11 (-0.52, 0.87) | 0.39 (0.35, 0.50) | 0.44 (0.37, 0.54) |
| Waist circumference z score | -0.55 (-0.86, 0.00) | -0.46 (-0.85, 0.1) | 0.27 (0.23, 0.35) | 0.53 (0.49, 0.59) |
| Skinfold thickness (Triceps & Subscapular) | 16.27 (13.73, 20.93) | 14.07 (11.25, 16.6) | 0.15 (0.11, 0.23) | 0.11 (0.06, 0.15) |
| Fat mass, percentage of total weight (%) | 27.19 (23.03, 32.84) | 22.23 (17.56, 25.71) | 0.58 (0.48, 0.70) | 0.43 (0.32, 0.51) |
| HDL, mg/L | 54.14 (50.27, 65.74) | 54.14 (47.37, 61.87) | 0.65 (0.50, 0.70) | 0.68 (0.59, 0.76) |
| Non-HDL, mg/L | 100.54 (85.07, 116.01) | 92.81 (81.21, 108.28) | 0.37 (0.27, 0.46) | 0.34 (0.27, 0.44) |
| Triglycerides, mg/L | 77.94 (61.11, 98.31) | 77.06 (59.56, 110.49) | 0.20 (0.14, 0.28) | 0.13 (0.08, 0.23) |
| Leptin, pg/ml | 10.06 (9.60, 11.09) | 9.2 (8.34, 9.98) | 0.55 (0.47, 0.72) | 0.5 (0.38, 0.61) |
| Adiponectin, pg/ml | 24.61 (24.03, 24.82) | 24.57 (24.21, 24.84) | 0.21 (0.17, 0.32) | 0.31 (0.22, 0.43) |
| *Immune/Inflammatory system* |  |  |  |  |
| CRP, pg/ml | 18.48 (17.69, 20.24) | 18.41 (17.72, 19.52) | 0.43 (0.34, 0.64) | 0.48 (0.4, 0.6) |
| IL-1B, pg/ml | 3.50 (3.15, 3.96) | 3.03 (2.69, 3.39) | 0.33 (0.25, 0.43) | 0.24 (0.17, 0.32) |
| IL-6, pg/ml | 3.36 (3.04, 3.74) | 3.06 (2.7, 3.34) | 0.36 (0.28, 0.47) | 0.27 (0.19, 0.33) |
| IL-8, pg/ml | 6.54 (6.35, 6.76) | 6.58 (6.38, 6.69) | 0.23 (0.19, 0.29) | 0.22 (0.16, 0.25) |
| IL-10, pg/ml | 3.24 (2.37, 3.80) | 3.39 (2.66, 4.03) | 0.62 (0.56, 0.71) | 0.59 (0.53, 0.66) |
| TNF-a, pg/ml | 5.01 (4.89, 5.21) | 5 (4.9, 5.13) | 0.19 (0.17, 0.24) | 0.21 (0.19, 0.24) |
| *Neuroendocrine system* |  |  |  |  |
| Cortisol production, µg/µmol creatinine, log10 transformed, median, [12.5^th^, 85.5^th^] | -0.13 (-0.35, 0.06) | -0.14 (-0.34, 0.03) | 0.13 (0.03, 0.27) | 0.2 (0.07, 0.37) |

Abbreviations: HDL, high-density lipoprotein cholesterol; CRP, C-reactive protein; IL, interleukin; TNF, Tumor necrosis factor.

^1^For the continuous allostatic load score, sex-stratified population distributions of biomarkers were rescaled to distributions between 0 and 1.

**eTable 3. Percentiles of biomarkers of allostatic load grouped in four physiological systems in each cohort - continued**

| Biomarkers | Distributional cut-off points  Median, percentiles [25^th^, 75^th^] | | Continuous score  Median, percentiles [25^th^, 75^th^] | |
| --- | --- | --- | --- | --- |
|  | Girls | Boys | Girls | Boys |
| EDEN, France |  |  |  |  |
| *Cardiovascular system* |  |  |  |  |
| Systolic blood pressure z score | 0.13 (-0.33, 0.55) | 0.05 (-0.29, 0.53) | 0.39 (0.33, 0.46) | 0.3 (0.26, 0.36) |
| Diastolic blood pressure z score | -0.11 (-0.59, 0.35) | -0.29 (-0.57, -0.02) | 0.29 (0.21, 0.37) | 0.21 (0.17, 0.25) |
| Pulse rate z score | 0.10 (-0.47, 0.84) | -0.08 (-0.66, 0.68) | 0.40 (0.32, 0.51) | 0.55 (0.46, 0.66) |
| *Metabolic system* |  |  |  |  |
| BMI z score | 0.27 (-0.42, 1.10) | 0.05 (-0.53, 1.35) | 0.42 (0.32, 0.55) | 0.44 (0.37, 0.59) |
| Waist circumference z score | -0.12 (-0.94, 0.35) | -0.45 (-0.93, 0.29) | 0.34 (0.21, 0.41) | 0.54 (0.49, 0.61) |
| Skinfold thickness (Triceps & Subscapular) | 22.33 (16.46, 29.88) | 17.33 (12.17, 26.33) | 0.26 (0.15, 0.39) | 0.16 (0.08, 0.31) |
| Fat mass, percentage of total weight (%) | 25.54 (21.16, 32.44) | 22.35 (18.47, 31.06) | 0.54 (0.44, 0.69) | 0.43 (0.34, 0.63) |
| HDL, mg/L | 58.01 (54.14, 61.87) | 61.87 (54.14, 73.47) | 0.60 (0.55, 0.65) | 0.59 (0.45, 0.68) |
| Non-HDL, mg/L | 110.21 (92.81, 123.74) | 100.54 (88.94, 119.88) | 0.43 (0.32, 0.51) | 0.39 (0.32, 0.51) |
| Triglycerides, mg/L | 79.71 (63.99, 108.72) | 80.6 (60.23, 102.74) | 0.21 (0.15, 0.32) | 0.14 (0.08, 0.2) |
| Leptin, pg/ml | 10.65 (10.10, 11.30) | 10.33 (9.42, 11.21) | 0.64 (0.55, 0.75) | 0.66 (0.53, 0.78) |
| Adiponectin, pg/ml | 24.50 (24.20, 24.81) | 24.6 (24.31, 24.73) | 0.23 (0.17, 0.29) | 0.3 (0.26, 0.4) |
| *Immune/Inflammatory system* |  |  |  |  |
| CRP, pg/ml | 19.76 (18.13, 21.14) | 19.43 (17.78, 20.89) | 0.59 (0.39, 0.75) | 0.59 (0.41, 0.74) |
| IL-1B, pg/ml | 4.01 (3.53, 5.03) | 3.38 (2.89, 4.67) | 0.44 (0.33, 0.67) | 0.31 (0.21, 0.58) |
| IL-6, pg/ml | 3.89 (3.34, 4.46) | 3.34 (2.88, 3.99) | 0.51 (0.36, 0.66) | 0.33 (0.23, 0.48) |
| IL-8, pg/ml | 6.52 (6.34, 6.71) | 6.38 (6.22, 6.58) | 0.23 (0.18, 0.27) | 0.16 (0.12, 0.22) |
| IL-10, pg/ml | 2.26 (1.76, 3.24) | 2.26 (1.14, 2.91) | 0.72 (0.62, 0.77) | 0.7 (0.64, 0.81) |
| TNF-a, pg/ml | 4.90 (4.75, 5.04) | 4.83 (4.62, 5) | 0.17 (0.14, 0.20) | 0.18 (0.13, 0.21) |
| *Neuroendocrine system* |  |  |  |  |
| Cortisol production, µg/µmol creatinine, log10 transformed, median, [12.5^th^, 85.5^th^] | -0.69 (-1.01, -0.44) | -0.73 (-0.99, -0.52) | 0.46 (0.22, 0.72) | 0.38 (0.16, 0.62) |

Abbreviations: HDL, high-density lipoprotein cholesterol; CRP, C-reactive protein; IL, interleukin; TNF, Tumor necrosis factor.

^1^For the continuous allostatic load score, sex-stratified population distributions of biomarkers were rescaled to distributions between 0 and 1.

**eTable 3. Percentiles of biomarkers of allostatic load grouped in four physiological systems in each cohort - continued**

| Biomarkers | Distributional cut-off points  Median, percentiles [25^th^, 75^th^] | | Continuous score  Median, percentiles [25^th^, 75^th^] | |
| --- | --- | --- | --- | --- |
|  | Girls | Boys | Girls | Boys |
| INMA, Spain |  |  |  |  |
| *Cardiovascular system* |  |  |  |  |
| Systolic blood pressure z score | 0.21 (-0.34, 0.97) | 0.46 (-0.24, 1.01) | 0.41 (0.32, 0.52) | 0.35 (0.26, 0.42) |
| Diastolic blood pressure z score | 0.05 (-0.40, 0.54) | -0.12 (-0.42, 0.5) | 0.32 (0.25, 0.40) | 0.23 (0.19, 0.32) |
| Pulse rate z score | 0.25 (-0.36, 0.92) | 0.11 (-0.56, 0.94) | 0.42 (0.33, 0.52) | 0.58 (0.48, 0.7) |
| *Metabolic system* |  |  |  |  |
| BMI z score | 0.58 (-0.02, 1.57) | 0.94 (-0.26, 1.87) | 0.47 (0.38, 0.62) | 0.54 (0.4, 0.66) |
| Waist circumference z score | 0.13 (-0.44, 1.04) | -0.1 (-0.53, 0.81) | 0.37 (0.29, 0.51) | 0.57 (0.53, 0.67) |
| Skinfold thickness (Triceps & Subscapular) | 21.77 (16.50, 28.03) | 16.87 (12.93, 25.9) | 0.25 (0.15, 0.36) | 0.15 (0.09, 0.3) |
| Fat mass, percentage of total weight (%) | 26.53 (21.29, 32.34) | 23.99 (18.44, 30.91) | 0.56 (0.44, 0.69) | 0.47 (0.34, 0.63) |
| HDL, mg/L | 58.01 (50.27, 65.74) | 58.01 (50.27, 69.61) | 0.60 (0.50, 0.70) | 0.64 (0.5, 0.73) |
| Non-HDL, mg/L | 104.41 (91.84, 119.88) | 100.54 (85.07, 108.28) | 0.39 (0.31, 0.49) | 0.39 (0.29, 0.44) |
| Triglycerides, mg/L | 80.60 (63.77, 112.26) | 74.4 (58.01, 102.74) | 0.21 (0.15, 0.33) | 0.12 (0.08, 0.2) |
| Leptin, pg/ml | 10.63 (10.00, 11.35) | 10.09 (9.54, 11.19) | 0.64 (0.54, 0.76) | 0.62 (0.55, 0.78) |
| Adiponectin, pg/ml | 24.61 (24.30, 24.85) | 24.54 (24.18, 24.75) | 0.21 (0.16, 0.27) | 0.32 (0.25, 0.44) |
| *Immune/Inflammatory system* |  |  |  |  |
| CRP, pg/ml | 19.86 (18.85, 20.95) | 19.72 (18.6, 20.81) | 0.60 (0.48, 0.73) | 0.62 (0.5, 0.74) |
| IL-1B, pg/ml | 3.96 (3.50, 5.01) | 3.54 (3.06, 4.45) | 0.43 (0.33, 0.66) | 0.35 (0.25, 0.53) |
| IL-6, pg/ml | 3.72 (3.41, 4.44) | 3.55 (3.14, 4.12) | 0.46 (0.38, 0.66) | 0.38 (0.29, 0.51) |
| IL-8, pg/ml | 6.50 (6.33, 6.64) | 6.45 (6.33, 6.58) | 0.22 (0.18, 0.26) | 0.18 (0.15, 0.22) |
| IL-10, pg/ml | 2.10 (1.26, 3.26) | 1.83 (1.25, 2.69) | 0.74 (0.61, 0.83) | 0.74 (0.66, 0.8) |
| TNF-a, pg/ml | 4.92 (4.74, 5.07) | 4.9 (4.67, 4.96) | 0.18 (0.14, 0.21) | 0.19 (0.14, 0.2) |
| *Neuroendocrine system* |  |  |  |  |
| Cortisol production, µg/µmol creatinine, log10 transformed, median, [12.5^th^, 85.5^th^] | -0.70 (-0.95, -0.47) | -0.74 (-1.03, -0.52) | 0.47 (0.23, 0.67) | 0.39 (0.15, 0.66) |

Abbreviations: HDL, high-density lipoprotein cholesterol; CRP, C-reactive protein; IL, interleukin; TNF, Tumor necrosis factor.

^1^For the continuous allostatic load score, sex-stratified population distributions of biomarkers were rescaled to distributions between 0 and 1.

**eTable 3. Percentiles of biomarkers of allostatic load grouped in four physiological systems in each cohort - continued**

| Biomarkers | Distributional cut-off points  Median, percentiles [25^th^, 75^th^] | | Continuous score  Median, percentiles [25^th^, 75^th^] | |
| --- | --- | --- | --- | --- |
|  | Girls | Boys | Girls | Boys |
| KANC, Lithuania |  |  |  |  |
| *Cardiovascular system* |  |  |  |  |
| Systolic blood pressure z score | -0.49 (-0.92, 0.11) | -0.77 (-1.06, -0.18) | 0.30 (0.23, 0.39) | 0.2 (0.16, 0.27) |
| Diastolic blood pressure z score | -0.06 (-0.34, 0.42) | -0.08 (-0.39, 0.24) | 0.30 (0.26, 0.38) | 0.24 (0.2, 0.28) |
| Pulse rate z score | 0.16 (-0.47, 0.80) | 0.29 (-0.19, 0.77) | 0.41 (0.32, 0.50) | 0.6 (0.53, 0.67) |
| *Metabolic system* |  |  |  |  |
| BMI z score | 0.62 (-0.33, 1.32) | 0.37 (-0.21, 1.2) | 0.48 (0.33, 0.58) | 0.48 (0.41, 0.58) |
| Waist circumference z score | 0.01 (-0.51, 0.54) | -0.17 (-0.48, 0.32) | 0.36 (0.28, 0.44) | 0.57 (0.53, 0.62) |
| Skinfold thickness (Triceps & Subscapular) | 17.20 (13.77, 21.83) | 13.27 (11.8, 15.6) | 0.17 (0.11, 0.25) | 0.09 (0.07, 0.13) |
| Fat mass, percentage of total weight (%) | 23.98 (20.93, 29.47) | 21.12 (17.88, 25.14) | 0.50 (0.44, 0.63) | 0.4 (0.32, 0.49) |
| HDL, mg/L | 58.01 (50.27, 61.87) | 58.01 (50.27, 65.74) | 0.60 (0.55, 0.70) | 0.64 (0.55, 0.73) |
| Non-HDL, mg/L | 104.41 (88.94, 120.84) | 100.54 (85.07, 116.01) | 0.39 (0.29, 0.49) | 0.39 (0.29, 0.49) |
| Triglycerides, mg/L | 77.50 (62.66, 107.83) | 75.28 (56.68, 95.66) | 0.20 (0.14, 0.32) | 0.13 (0.07, 0.18) |
| Leptin, pg/ml | 9.98 (9.25, 10.87) | 9.24 (8.4, 10.36) | 0.53 (0.41, 0.68) | 0.51 (0.39, 0.66) |
| Adiponectin, pg/ml | 24.69 (24.32, 24.99) | 24.54 (24.2, 24.81) | 0.19 (0.13, 0.27) | 0.32 (0.23, 0.43) |
| *Immune/Inflammatory system* |  |  |  |  |
| CRP, pg/ml | 19.50 (18.23, 20.77) | 18.51 (17.36, 19.65) | 0.55 (0.40, 0.71) | 0.49 (0.36, 0.61) |
| IL-1B, pg/ml | 3.51 (3.01, 4.19) | 3.03 (2.77, 3.43) | 0.33 (0.22, 0.48) | 0.24 (0.19, 0.32) |
| IL-6, pg/ml | 3.56 (3.05, 4.00) | 3.06 (2.78, 3.43) | 0.42 (0.28, 0.54) | 0.27 (0.21, 0.35) |
| IL-8, pg/ml | 6.54 (6.38, 6.69) | 6.46 (6.35, 6.61) | 0.23 (0.19, 0.27) | 0.19 (0.15, 0.23) |
| IL-10, pg/ml | 2.11 (1.36, 2.81) | 2.04 (1.38, 3.04) | 0.74 (0.66, 0.81) | 0.72 (0.62, 0.79) |
| TNF-a, pg/ml | 4.93 (4.79, 5.10) | 4.9 (4.75, 4.97) | 0.18 (0.15, 0.21) | 0.19 (0.16, 0.2) |
| *Neuroendocrine system* |  |  |  |  |
| Cortisol production, µg/µmol creatinine, log10 transformed, median, [12.5^th^, 85.5^th^] | -0.50 (-0.79, -0.22) | -0.53 (-0.81, -0.22) | 0.28 (0.08, 0.53) | 0.23 (0.06, 0.46) |

Abbreviations: HDL, high-density lipoprotein cholesterol; CRP, C-reactive protein; IL, interleukin; TNF, Tumor necrosis factor.

^1^For the continuous allostatic load score, sex-stratified population distributions of biomarkers were rescaled to distributions between 0 and 1.

**eTable 3. Percentiles of biomarkers of allostatic load grouped in four physiological systems in each cohort - continued**

| Biomarkers | Distributional cut-off points  Median, percentiles [25^th^, 75^th^] | | | Continuous score  Median, percentiles [25^th^, 75^th^] | | |
| --- | --- | --- | --- | --- | --- | --- |
|  | Girls | Boys | Girls | | Boys |  |
| MoBa, Norway |  |  |  | |  |  |
| *Cardiovascular system* |  |  |  | |  |  |
| Systolic blood pressure z score | -0.29 (-0.76, 0.40) | -0.3 (-0.81, 0.32) | 0.33 (0.26, 0.44) | | 0.25 (0.19, 0.33) |  |
| Diastolic blood pressure z score | -0.28 (-0.79, 0.00) | -0.44 (-0.87, -0.04) | 0.27 (0.18, 0.31) | | 0.19 (0.13, 0.24) |  |
| Pulse rate z score | -0.51 (-1.19, 0.10) | -0.24 (-0.98, 0.21) | 0.31 (0.21, 0.40) | | 0.52 (0.41, 0.59) |  |
| *Metabolic system* |  |  |  | |  |  |
| BMI z score | 0.32 (-0.24, 1.07) | -0.11 (-0.53, 0.5) | 0.43 (0.35, 0.54) | | 0.42 (0.37, 0.49) |  |
| Waist circumference z score | -0.39 (-0.76, 0.25) | -0.51 (-0.76, -0.16) | 0.30 (0.24, 0.39) | | 0.53 (0.5, 0.57) |  |
| Skinfold thickness (Triceps & Subscapular) | 15.85 (13.43, 19.84) | 12.07 (10.86, 13.84) | 0.14 (0.10, 0.21) | | 0.08 (0.06, 0.1) |  |
| Fat mass, percentage of total weight (%) | 18.16 (14.67, 23.76) | 12.89 (10.48, 17.56) | 0.37 (0.30, 0.50) | | 0.21 (0.15, 0.32) |  |
| HDL, mg/L | 61.87 (54.14, 69.61) | 61.87 (52.2, 69.61) | 0.55 (0.45, 0.65) | | 0.59 (0.5, 0.7) |  |
| Non-HDL, mg/L | 108.28 (92.81, 123.74) | 96.68 (85.07, 116.01) | 0.41 (0.32, 0.51) | | 0.37 (0.29, 0.49) |  |
| Triglycerides, mg/L | 74.84 (59.78, 105.62) | 69.97 (53.58, 90.34) | 0.19 (0.13, 0.31) | | 0.11 (0.07, 0.17) |  |
| Leptin, pg/ml | 10.55 (9.90, 11.18) | 9.13 (8.23, 10.1) | 0.63 (0.52, 0.73) | | 0.49 (0.37, 0.63) |  |
| Adiponectin, pg/ml | 24.53 (24.21, 24.85) | 24.58 (24.14, 24.83) | 0.22 (0.16, 0.29) | | 0.31 (0.23, 0.45) |  |
| *Immune/Inflammatory system* |  |  |  | |  |  |
| CRP, pg/ml | 19.18 (18.39, 20.28) | 18.14 (16.66, 19.73) | 0.52 (0.42, 0.65) | | 0.45 (0.29, 0.62) |  |
| IL-1B, pg/ml | 3.64 (3.23, 4.13) | 2.91 (2.63, 3.3) | 0.36 (0.27, 0.47) | | 0.22 (0.16, 0.3) |  |
| IL-6, pg/ml | 3.40 (3.10, 3.78) | 3.02 (2.78, 3.29) | 0.37 (0.29, 0.48) | | 0.26 (0.2, 0.32) |  |
| IL-8, pg/ml | 6.42 (6.26, 6.54) | 6.43 (6.26, 6.54) | 0.20 (0.16, 0.23) | | 0.18 (0.13, 0.21) |  |
| IL-10, pg/ml | 2.15 (1.42, 3.11) | 2.12 (1.42, 2.76) | 0.73 (0.63, 0.81) | | 0.71 (0.65, 0.78) |  |
| TNF-a, pg/ml | 4.83 (4.64, 4.95) | 4.91 (4.75, 5) | 0.16 (0.11, 0.18) | | 0.19 (0.16, 0.21) |  |
| *Neuroendocrine system* |  |  |  | |  |  |
| Cortisol production, µg/µmol creatinine, log10 transformed, median, [12.5^th^, 85.5^th^] | -0.63 (-0.94, -0.39) | -0.64 (-0.95, -0.41) | 0.40 (0.16, 0.67) | | 0.29 (0.07, 0.57) |  |

Abbreviations: HDL, high-density lipoprotein cholesterol; CRP, C-reactive protein; IL, interleukin; TNF, Tumor necrosis factor.

^1^For the continuous allostatic load score, sex-stratified population distributions of biomarkers were rescaled to distributions between 0 and 1.

**eTable 3. Percentiles of biomarkers of allostatic load grouped in four physiological systems in each cohort - continued**

| Biomarkers | Distributional cut-off points  Median, percentiles [25^th^, 75^th^] | | Continuous score  Median, percentiles [25^th^, 75^th^] | |
| --- | --- | --- | --- | --- |
|  | Girls | Boys | Girls | Boys |
| Rhea, Greece |  |  |  |  |
| *Cardiovascular system* |  |  |  |  |
| Systolic blood pressure z score | -0.16 (-0.72, 0.37) | -0.5 (-0.95, 0.01) | 0.35 (0.27, 0.43) | 0.23 (0.17, 0.29) |
| Diastolic blood pressure z score | -0.14 (-0.65, 0.20) | -0.42 (-0.67, -0.06) | 0.29 (0.20, 0.34) | 0.19 (0.16, 0.24) |
| Pulse rate z score | 0.16 (-0.40, 0.80) | 0.05 (-0.38, 0.63) | 0.41 (0.33, 0.50) | 0.57 (0.5, 0.65) |
| *Metabolic system* |  |  |  |  |
| BMI z score | 0.70 (-0.44, 1.25) | 0.46 (-0.32, 1.58) | 0.49 (0.32, 0.57) | 0.49 (0.39, 0.62) |
| Waist circumference z score | -0.01 (-0.44, 0.45) | 0.01 (-0.29, 0.88) | 0.35 (0.29, 0.42) | 0.58 (0.55, 0.68) |
| Skinfold thickness (Triceps & Subscapular) | 17.93 (14.50, 20.18) | 14.02 (11.9, 17.34) | 0.18 (0.12, 0.22) | 0.11 (0.07, 0.16) |
| Fat mass, percentage of total weight (%) | 25.17 (21.55, 27.01) | 21.66 (17.26, 26.21) | 0.53 (0.45, 0.57) | 0.41 (0.31, 0.52) |
| HDL, mg/L | 58.01 (50.27, 67.67) | 61.87 (54.14, 69.61) | 0.60 (0.48, 0.70) | 0.59 (0.5, 0.68) |
| Non-HDL, mg/L | 104.41 (87.01, 112.14) | 100.54 (88.94, 104.41) | 0.39 (0.28, 0.44) | 0.39 (0.32, 0.41) |
| Triglycerides, mg/L | 69.97 (56.68, 87.24) | 63.33 (52.03, 88.13) | 0.17 (0.12, 0.24) | 0.09 (0.06, 0.16) |
| Leptin, pg/ml | 10.62 (9.79, 11.40) | 9.63 (8.58, 10.32) | 0.64 (0.50, 0.77) | 0.56 (0.42, 0.66) |
| Adiponectin, pg/ml | 24.49 (24.04, 24.76) | 24.47 (24.14, 24.83) | 0.23 (0.18, 0.32) | 0.35 (0.23, 0.45) |
| *Immune/Inflammatory system* |  |  |  |  |
| CRP, pg/ml | 19.63 (17.75, 21.10) | 19.06 (17.83, 20.49) | 0.57 (0.35, 0.75) | 0.55 (0.42, 0.7) |
| IL-1B, pg/ml | 3.60 (3.21, 4.16) | 3.19 (2.78, 3.71) | 0.35 (0.26, 0.47) | 0.27 (0.19, 0.38) |
| IL-6, pg/ml | 3.63 (3.29, 3.96) | 3.29 (3, 3.67) | 0.44 (0.34, 0.53) | 0.32 (0.26, 0.41) |
| IL-8, pg/ml | 6.58 (6.44, 6.70) | 6.54 (6.44, 6.66) | 0.24 (0.21, 0.27) | 0.21 (0.18, 0.24) |
| IL-10, pg/ml | 2.86 (1.96, 3.83) | 2.89 (2.16, 3.6) | 0.66 (0.56, 0.75) | 0.64 (0.57, 0.71) |
| TNF-a, pg/ml | 5.04 (4.93, 5.22) | 4.97 (4.84, 5.17) | 0.20 (0.18, 0.24) | 0.2 (0.18, 0.25) |
| *Neuroendocrine system* |  |  |  |  |
| Cortisol production, µg/µmol creatinine, log10 transformed, median, [12.5^th^, 85.5^th^] | -0.14 (-0.70, 0.17) | -0.1 (-0.72, 0.12) | 0.27 (0.06, 0.52) | 0.29 (0.16, 0.55) |

Abbreviations: HDL, high-density lipoprotein cholesterol; CRP, C-reactive protein; IL, interleukin; TNF, Tumor necrosis factor.

^1^For the continuous allostatic load score, sex-stratified population distributions of biomarkers were rescaled to distributions between 0 and 1.

**eTable 4. Characteristics of study population in each cohort**

| Characteristics | N (%), mean (SD) or median [25^th^, 75^th^] |
| --- | --- |
| BiB, UK |  |
| *Child* |  |
| Age, years | 6.6 [6.4, 6.7] |
| Sex (Girls) | 53 (43.1%) |
| Ancestry (European) | 52 (42.3%) |
| Moderate-to-vigorous physical activity, mins/day | 51.4 [19.3, 85.7] |
| Sedentary time, mins/day | 231.4 [142.9, 295.7] |
| Exposure to second-hand smoking (Yes) | 25 (20.4%) |
| *Parents* |  |
| Age of mothers, years | 29.2 (5.5) |
| Smoking during pregnancy (Yes) | 13 (10.8%) |
| Alcohol drinking during pregnancy (Yes) | 50 (40.7%) |
| Parity |  |
| Nulliparous | 47 (38.4%) |
| Primiparous | 37 (30.0%) |
| Multiparous | 39 (31.6%) |
| Marital and habitation status |  |
| Living with the father | 108 (87.8%) |
| Other situation | 15 (12.2%) |
| Smoking status of parents |  |
| Neither | 88 (71.9%) |
| One | 30 (24.7%) |
| Both | 4 (3.4%) |

**eTable 4. Characteristics of study population in each cohort - continued**

| Characteristics | N (%), mean (SD) or median [25^th^, 75^th^] |
| --- | --- |
| BiB, UK |  |
| *Socioeconomic status* |  |
| Maternal educational level |  |
| Low | 54 (43.9%) |
| Middle | 24 (19.9%) |
| High | 45 (36.3%) |
| Paternal educational level |  |
| Low | 57 (46.5%) |
| Middle | 21 (17.1%) |
| High | 45 (36.4%) |
| Family Affluence Scale |  |
| Low | 31 (25.2%) |
| Middle | 57 (46.3%) |
| High | 35 (28.5%) |
| Parental national origin |  |
| None or one native | 68 (55.6%) |
| Both native | 55 (44.4%) |
| *Allostatic load* |  |
| Count-based allostatic load score | 4.0 [2.0, 6.0] |
| Continuous allostatic load score | 6.9 [6.4, 7.8] |

**eTable 4. Characteristics of study population in each cohort - continued**

| Characteristics | N (%), mean (SD) or median [25^th^, 75^th^] |
| --- | --- |
| EDEN, France |  |
| *Child* |  |
| Age, years | 10.8 [10.3, 11.1] |
| Sex (Girls) | 54 (43.9%) |
| Ancestry (European) | 123 (100.0%) |
| Moderate-to-vigorous physical activity, mins/day | 17.1 [5.7, 34.3] |
| Sedentary time, mins/day | 179.3 [134.3, 229.3] |
| Exposure to second-hand smoking (Yes) | 33 (27.1%) |
| *Parents* |  |
| Age of mothers, years | 30.9 (4.9) |
| Smoking during pregnancy (Yes) | 27 (22.0%) |
| Alcohol drinking during pregnancy (Yes) | 52 (42.2%) |
| Parity |  |
| Nulliparous | 56 (45.5%) |
| Primiparous | 44 (35.8%) |
| Multiparous | 23 (18.7%) |
| Marital and habitation status |  |
| Living with the father | 120 (97.6%) |
| Other situation | 3 (2.4%) |
| Smoking status of parents |  |
| Neither | 70 (56.9%) |
| One | 34 (27.6%) |
| Both | 19 (15.4%) |

**eTable 4. Characteristics of study population in each cohort - continued**

| Characteristics | N (%), mean (SD) or median [25^th^, 75^th^] |
| --- | --- |
| EDEN, France |  |
| *Socioeconomic status* |  |
| Maternal educational level |  |
| Low | 8 (6.5%) |
| Middle | 47 (38.0%) |
| High | 68 (55.5%) |
| Paternal educational level |  |
| Low | 15 (12.4%) |
| Middle | 58 (47.3%) |
| High | 50 (40.3%) |
| Family Affluence Scale |  |
| Low | 0 (0.0%) |
| Middle | 29 (23.6%) |
| High | 94 (76.4%) |
| Parental national origin |  |
| None or one native | 8 (6.8%) |
| Both native | 115 (93.2%) |
| *Allostatic load* |  |
| Count-based allostatic load score | 5.0 [2.5, 8.0] |
| Continuous allostatic load score | 7.6 [6.8, 8.8] |

**eTable 4. Characteristics of study population in each cohort - continued**

| Characteristics | N (%), mean (SD) or median [25^th^, 75^th^] |
| --- | --- |
| INMA, Spain |  |
| *Child* |  |
| Age, years | 8.8 [8.4, 9.2] |
| Sex (Girls) | 76 (41.5%) |
| Ancestry (European) | 183 (100.0%) |
| Moderate-to-vigorous physical activity, mins/day | 45.7 [25.7, 75.0] |
| Sedentary time, mins/day | 192.9 [155.7, 254.3] |
| Exposure to second-hand smoking (Yes) | 61 (33.4%) |
| *Parents* |  |
| Age of mothers, years | 32.1 (3.9) |
| Smoking during pregnancy (Yes) | 48 (26.0%) |
| Alcohol drinking during pregnancy (Yes) | 36 (19.7%) |
| Parity |  |
| Nulliparous | 96 (52.3%) |
| Primiparous | 77 (42.3%) |
| Multiparous | 10 (5.5%) |
| Marital and habitation status |  |
| Living with the father | 182 (99.5%) |
| Other situation | 1 (0.5%) |
| Smoking status of parents |  |
| Neither | 105 (57.2%) |
| One | 51 (28.0%) |
| Both | 27 (14.8%) |

**eTable 4. Characteristics of study population in each cohort - continued**

| Characteristics | N (%), mean (SD) or median [25^th^, 75^th^] |
| --- | --- |
| INMA, Spain |  |
| *Socioeconomic status* |  |
| Maternal educational level |  |
| Low | 43 (23.3%) |
| Middle | 76 (41.7%) |
| High | 64 (35.0%) |
| Paternal educational level |  |
| Low | 60 (32.7%) |
| Middle | 79 (43.3%) |
| High | 44 (24.0%) |
| Family Affluence Scale |  |
| Low | 10 (5.5%) |
| Middle | 70 (38.4%) |
| High | 103 (56.1%) |
| Parental national origin |  |
| None or one native | 9 (4.9%) |
| Both native | 174 (95.1%) |
| *Allostatic load* |  |
| Count-based allostatic load score | 5.0 [3.0, 9.5] |
| Continuous allostatic load score | 7.9 [7.1, 9.1] |

**eTable 4. Characteristics of study population in each cohort - continued**

| Characteristics | N (%), mean (SD) or median [25^th^, 75^th^] |
| --- | --- |
| KANC, Lithuania |  |
| *Child* |  |
| Age, years | 6.3 [6.1, 6.8] |
| Sex (Girls) | 80 (45.2%) |
| Ancestry (European) | 177 (100.0%) |
| Moderate-to-vigorous physical activity, mins/day | 68.6 [19.3, 95.0] |
| Sedentary time, mins/day | 325.7 [248.6, 437.1] |
| Exposure to second-hand smoking (Yes) | 78 (43.9%) |
| *Parents* |  |
| Age of mothers, years | 29.2 (5.0) |
| Smoking during pregnancy (Yes) | 11 (6.4%) |
| Alcohol drinking during pregnancy (Yes) | 17 (9.4%) |
| Parity |  |
| Nulliparous | 73 (41.0%) |
| Primiparous | 53 (30.1%) |
| Multiparous | 51 (28.9%) |
| Marital and habitation status |  |
| Living with the father | 151 (85.5%) |
| Other situation | 26 (14.5%) |
| Smoking status of parents |  |
| Neither | 90 (50.6%) |
| One | 64 (36.1%) |
| Both | 24 (13.3%) |

**eTable 4. Characteristics of study population in each cohort - continued**

| Characteristics | N (%), mean (SD) or median [25^th^, 75^th^] |
| --- | --- |
| KANC, Lithuania |  |
| *Socioeconomic status* |  |
| Maternal educational level |  |
| Low | 8 (4.5%) |
| Middle | 63 (35.6%) |
| High | 106 (60.0%) |
| Paternal educational level |  |
| Low | 13 (7.4%) |
| Middle | 86 (48.6%) |
| High | 78 (44.1%) |
| Family Affluence Scale |  |
| Low | 27 (15.3%) |
| Middle | 90 (51.1%) |
| High | 59 (33.6%) |
| Parental national origin |  |
| None or one native | 9 (5.0%) |
| Both native | 168 (95.0%) |
| *Allostatic load* |  |
| Count-based allostatic load score | 3.0 [2.0, 6.0] |
| Continuous allostatic load score | 7.1 [6.4, 7.9] |

**eTable 4. Characteristics of study population in each cohort - continued**

| Characteristics | N (%), mean (SD) or median [25^th^, 75^th^] |
| --- | --- |
| MoBa, Norway |  |
| *Child* |  |
| Age, years | 8.6 [8.2, 8.8] |
| Sex (Girls) | 88 (45.8%) |
| Ancestry (European) | 184 (95.8%) |
| Moderate-to-vigorous physical activity, mins/day | 64.3 [36.4, 92.6] |
| Sedentary time, mins/day | 177.5 [137.8, 257.1] |
| Exposure to second-hand smoking (Yes) | 36 (18.8%) |
| *Parents* |  |
| Age of mothers, years | 32.8 (3.6) |
| Smoking during pregnancy (Yes) | 8 (4.0%) |
| Alcohol drinking during pregnancy (Yes) | 96 (50.0%) |
| Parity |  |
| Nulliparous | 84 (44.0%) |
| Primiparous | 82 (42.5%) |
| Multiparous | 26 (13.5%) |
| Marital and habitation status |  |
| Living with the father | 188 (98.0%) |
| Other situation | 4 (2.0%) |
| Smoking status of parents |  |
| Neither | 175 (91.1%) |
| One | 13 (6.8%) |
| Both | 4 (2.1%) |

**eTable 4. Characteristics of study population in each cohort - continued**

| Characteristics | N (%), mean (SD) or median [25^th^, 75^th^] |
| --- | --- |
| MoBa, Norway |  |
| *Socioeconomic status* |  |
| Maternal educational level |  |
| Low | 0 (0.0%) |
| Middle | 37 (19.1%) |
| High | 155 (80.9%) |
| Paternal educational level |  |
| Low | 0 (0.0%) |
| Middle | 45 (23.5%) |
| High | 147 (76.5%) |
| Family Affluence Scale |  |
| Low | 2 (1.0%) |
| Middle | 54 (28.1%) |
| High | 136 (70.8%) |
| Parental national origin |  |
| None or one native | 36 (18.6%) |
| Both native | 156 (81.4%) |
| *Allostatic load* |  |
| Count-based allostatic load score | 3.0 [2.0, 4.0] |
| Continuous allostatic load score | 6.7 [6.1, 7.5] |

**eTable 4. Characteristics of study population in each cohort - continued**

| Characteristics | N (%), mean (SD) or median [25^th^, 75^th^] |
| --- | --- |
| Rhea, Greece |  |
| *Child* |  |
| Age, years | 6.5 [6.4, 6.7] |
| Sex (Girls) | 55 (45.5%) |
| Ancestry (European) | 121 (100.0%) |
| Moderate-to-vigorous physical activity, mins/day | 25.7 [17.1, 38.6] |
| Sedentary time, mins/day | 210.0 [155.7, 287.1] |
| Exposure to second-hand smoking (Yes) | 81 (67.0%) |
| *Parents* |  |
| Age of mothers, years | 30.5 (4.7) |
| Smoking during pregnancy (Yes) | 25 (20.7%) |
| Alcohol drinking during pregnancy (Yes) | 39 (32.3%) |
| Parity |  |
| Nulliparous | 45 (37.3%) |
| Primiparous | 52 (43.0%) |
| Multiparous | 24 (19.7%) |
| Marital and habitation status |  |
| Living with the father | 119 (98.3%) |
| Other situation | 2 (1.7%) |
| Smoking status of parents |  |
| Neither | 50 (41.7%) |
| One | 48 (39.3%) |
| Both | 23 (19.0%) |

**eTable 4. Characteristics of study population in each cohort - continued**

| Characteristics | N (%), mean (SD) or median [25^th^, 75^th^] |
| --- | --- |
| Rhea, Greece |  |
| *Socioeconomic status* |  |
| Maternal educational level |  |
| Low | 3 (2.5%) |
| Middle | 74 (61.2%) |
| High | 44 (36.4%) |
| Paternal educational level |  |
| Low | 17 (14.2%) |
| Middle | 72 (59.3%) |
| High | 32 (26.4%) |
| Family Affluence Scale |  |
| Low | 15 (12.4%) |
| Middle | 62 (51.2%) |
| High | 44 (36.4%) |
| Parental national origin |  |
| None or one native | 5 (4.1%) |
| Both native | 116 (95.9%) |
| *Allostatic load* |  |
| Count-based allostatic load score | 4.0 [3.0, 5.0] |
| Continuous allostatic load score | 7.1 [6.5, 8.0] |

**eTable 5. Adjusted relative risk of count-based allostatic load score of each physiological system associated with road traffic noise levels**

|  | Relative risk | 95% CI |
| --- | --- | --- |
| Cardiovascular allostatic load score |  |  |
| Home |  |  |
| <55 dB | Reference | Reference |
| 55-59.9 dB | 1.23 | 0.98, 1.55 |
| 60-64.9 dB | 0.97 | 0.74, 1.27 |
| $\geq$65 dB | 0.90 | 0.65, 1.22 |
| School |  |  |
| <55 dB | Reference | Reference |
| 55-59.9 dB | 1.15 | 0.88, 1.51 |
| 60-64.9 dB | 1.15 | 0.82, 1.60 |
| $\geq$65 dB | 0.94 | 0.63, 1.40 |
| Metabolic allostatic load score |  |  |
| Home |  |  |
| <55 dB | Reference | Reference |
| 55-59.9 dB | 1.02 | 0.84, 1.25 |
| 60-64.9 dB | 0.90 | 0.71, 1.14 |
| $\geq$65 dB | 0.92 | 0.70, 1.20 |
| School |  |  |
| <55 dB | Reference | Reference |
| 55-59.9 dB | 1.08 | 0.85, 1.38 |
| 60-64.9 dB | 1.03 | 0.76, 1.39 |
| $\geq$65 dB | 0.94 | 0.66, 1.34 |

**eTable 5. Adjusted relative risk of count-based allostatic load score of each physiological system associated with road traffic noise levels - continued**

|  | Relative risk | 95% CI |
| --- | --- | --- |
| Immune/inflammatory allostatic load score |  |  |
| Home |  |  |
| <55 dB | Reference | Reference |
| 55-59.9 dB | 1.04 | 0.85, 1.26 |
| 60-64.9 dB | 0.86 | 0.68, 1.09 |
| $\geq$65 dB | 0.84 | 0.64, 1.08 |
| School |  |  |
| <55 dB | Reference | Reference |
| 55-59.9 dB | 0.93 | 0.73, 1.20 |
| 60-64.9 dB | 0.91 | 0.68, 1.23 |
| $\geq$65 dB | 0.77 | 0.54, 1.11 |
| Neuroendocrine load score |  |  |
| Home |  |  |
| <55 dB | Reference | Reference |
| 55-59.9 dB | 1.27 | 0.82, 1.97 |
| 60-64.9 dB | 1.38 | 0.84, 2.27 |
| $\geq$65 dB | 1.39 | 0.79, 2.44 |
| School |  |  |
| <55 dB | Reference | Reference |
| 55-59.9 dB | 1.19 | 0.71, 1.99 |
| 60-64.9 dB | 1.75 | 0.94, 3.29 |
| $\geq$65 dB | 1.25 | 0.58, 2.69 |

Abbreviations: dB, decibel. The negative binomial regression models were adjusted for cohort, child sex, age, ancestry, physical activity, sedentary behaviors, exposure to second-hand smoking at the 6-11 years follow-up, the family’s economic capital, maternal and paternal education, parental country of origin, maternal marital status, parity, alcohol drinking during pregnancy, and active smoking during pregnancy.

**eTable 6. Adjusted effect estimates of continuous allostatic load score of each physiological system associated with road traffic noise levels**

|  | β | 95% CI |
| --- | --- | --- |
| Cardiovascular allostatic load score |  |  |
| Home |  |  |
| <55 dB | Reference | Reference |
| 55-59.9 dB | 0.04 | -0.01, 0.10 |
| 60-64.9 dB | 0.01 | -0.06, 0.07 |
| $\geq$65 dB | -0.03 | -0.11, 0.05 |
| School |  |  |
| <55 dB | Reference | Reference |
| 55-59.9 dB | 0.08 | 0.00, 0.15 |
| 60-64.9 dB | 0.03 | -0.07, 0.12 |
| $\geq$65 dB | -0.01 | -0.12, 0.10 |
| Metabolic allostatic load score |  |  |
| Home |  |  |
| <55 dB | Reference | Reference |
| 55-59.9 dB | 0.05 | -0.11, 0.21 |
| 60-64.9 dB | -0.08 | -0.27, 0.11 |
| $\geq$65 dB | -0.01 | -0.23, 0.20 |
| School |  |  |
| <55 dB | Reference | Reference |
| 55-59.9 dB | 0.08 | -0.12, 0.27 |
| 60-64.9 dB | 0.03 | -0.22, 0.28 |
| $\geq$65 dB | -0.13 | -0.42, 0.17 |

**eTable 6. Adjusted effect estimates of continuous allostatic load score of each physiological system associated with road traffic noise levels - continued**

|  | β | 95% CI |
| --- | --- | --- |
| Cardiovascular allostatic load score |  |  |
| Home |  |  |
| <55 dB | Reference | Reference |
| 55-59.9 dB | 0.02 | -0.09, 0.12 |
| 60-64.9 dB | -0.03 | -0.15, 0.10 |
| $\geq$65 dB | -0.08 | -0.22, 0.06 |
| School |  |  |
| <55 dB | Reference | Reference |
| 55-59.9 dB | 0.02 | -0.11, 0.15 |
| 60-64.9 dB | -0.04 | -0.21, 0.12 |
| $\geq$65 dB | -0.19 | -0.38, 0.00 |
| Metabolic allostatic load score |  |  |
| Home |  |  |
| <55 dB | Reference | Reference |
| 55-59.9 dB | 0.02 | -0.02, 0.06 |
| 60-64.9 dB | 0.03 | -0.02, 0.08 |
| $\geq$65 dB | 0.00 | -0.05, 0.06 |
| School |  |  |
| <55 dB | Reference | Reference |
| 55-59.9 dB | 0.01 | -0.04, 0.06 |
| 60-64.9 dB | 0.06 | -0.01, 0.12 |
| $\geq$65 dB | 0.05 | -0.03, 0.12 |

Abbreviations: dB, decibel. The linear regression models were adjusted for cohort, child sex, age, ancestry, physical activity, sedentary behaviors, exposure to second-hand smoking at the 6-11 years follow-up, the family’s economic capital, maternal and paternal education, parental country of origin, maternal marital status, parity, alcohol drinking during pregnancy, and active smoking during pregnancy.

**eTable 7. Minimally adjusted relative risk of count-based allostatic load score associated with outdoor air pollutants and road traffic noise levels**

|  | Relative risk | 95% CI |
| --- | --- | --- |
| Outdoor air pollutants |  |  |
| NO_2_, 10 µg/m^3^ | 1.07 | 0.99, 1.16 |
| PM_2.5_, 5 µg/m^3^ | 1.16 | 1.00, 1.35 |
| PM_2.5_ absorbance, 1 10⁻⁵m⁻¹ | 1.17 | 0.96, 1.43 |
| PM_10_, 10 µg/m^3^ | 1.28 | 1.09, 1.51 |
| Road traffic noise |  |  |
| Home |  |  |
| <55 dB | Reference | Reference |
| 55-59.9 dB | 1.06 | 0.92, 1.22 |
| 60-64.9 dB | 0.93 | 0.78, 1.11 |
| $\geq$65 dB | 0.88 | 0.73, 1.07 |
| School |  |  |
| <55 dB | Reference | Reference |
| 55-59.9 dB | 1.02 | 0.86, 1.22 |
| 60-64.9 dB | 1.01 | 0.81, 1.27 |
| $\geq$65 dB | 0.86 | 0.67, 1.12 |

Abbreviations: CI, confidence interval; NO_2_, nitrogen dioxide; PM, particulate matter; dB, decibel. The negative binomial regression models were adjusted for cohort, child sex, age, and ancestry.

**eTable 8. Minimally adjusted effect estimates of continuous allostatic load score associated with outdoor air pollutants and road traffic noise levels**

|  | β | 95% CI |
| --- | --- | --- |
| Outdoor air pollutants |  |  |
| NO_2_, 10 µg/m^3^ | 0.16 | 0.01, 0.30 |
| PM_2.5_, 5 µg/m^3^ | 0.34 | 0.07, 0.61 |
| PM_2.5_ absorbance, 1 10⁻⁵m⁻¹ | 0.38 | 0.00, 0.75 |
| PM_10_, 10 µg/m^3^ | 0.56 | 0.27, 0.85 |
| Road traffic noise |  |  |
| Home |  |  |
| <55 dB | Reference | Reference |
| 55-59.9 dB | 0.10 | -0.15, 0.36 |
| 60-64.9 dB | -0.06 | -0.37, 0.24 |
| $\geq$65 dB | -0.18 | -0.53, 0.16 |
| School |  |  |
| <55 dB | Reference | Reference |
| 55-59.9 dB | 0.12 | -0.19, 0.44 |
| 60-64.9 dB | 0.02 | -0.39, 0.43 |
| $\geq$65 dB | -0.37 | -0.84, 0.11 |

Abbreviations: CI, confidence interval; NO_2_, nitrogen dioxide; PM, particulate matter; dB, decibel. The linear regression models were adjusted for cohort, child sex, age, ancestry, and ancestry.

**eTable 9. Adjusted relative risk of count-based allostatic load score associated with outdoor air pollutants and road traffic noise levels, when considering only high values of cortical production as ‘high risk’ in allostatic load scores**

|  | Relative risk | 95% CI |
| --- | --- | --- |
| Outdoor air pollutants |  |  |
| NO_2_, 10 µg/m^3^ | 1.16 | 1.00, 1.16 |
| PM_2.5_, 5 µg/m^3^ | 1.34 | 0.99, 1.34 |
| PM_2.5_ absorbance, 1 10⁻⁵m⁻¹ | 1.49 | 1.00, 1.49 |
| PM_10_, 10 µg/m^3^ | 1.51* | 1.09, 1.51* |
| Road traffic noise |  |  |
| Home |  |  |
| <55 dB | Reference | Reference |
| 55-59.9 dB | 1.22 | 0.92, 1.22 |
| 60-64.9 dB | 1.09 | 0.77, 1.09 |
| $\geq$65 dB | 1.09 | 0.74, 1.09 |
| School |  |  |
| <55 dB | Reference | Reference |
| 55-59.9 dB | 1.27 | 0.90, 1.27 |
| 60-64.9 dB | 1.30 | 0.84, 1.30 |
| $\geq$65 dB | 1.20 | 0.72, 1.20 |

Abbreviations: CI, confidence interval; NO_2_, nitrogen dioxide; PM, particulate matter; dB, decibel. The negative binomial regression models were adjusted for cohort, child sex, age, ancestry, physical activity, sedentary behaviors, exposure to second-hand smoking at the 6-11 years follow-up, the family’s economic capital, maternal and paternal education, parental country of origin, maternal marital status, parity, alcohol drinking during pregnancy, and active smoking during pregnancy.

*denotes the associations which remained statistically significant after multiple testing with five effective number of tests and corrected significance level at α = 0.01.

**eTable 10. Adjusted effect estimates of continuous allostatic load score associated with outdoor air pollutants and road traffic noise levels,** **when considering only high values of cortical production as ‘high risk’ in allostatic load scores**

|  | β | 95% CI |
| --- | --- | --- |
| Outdoor air pollutants |  |  |
| NO_2_, 10 µg/m^3^ | 0.18 | 0.04, 0.33 |
| PM_2.5_, 5 µg/m^3^ | 0.33 | 0.06, 0.61 |
| PM_2.5_ absorbance, 1 10⁻⁵m⁻¹ | 0.47 | 0.09, 0.86 |
| PM_10_, 10 µg/m^3^ | 0.57* | 0.28, 0.87* |
| Road traffic noise |  |  |
| Home |  |  |
| <55 dB | Reference | Reference |
| 55-59.9 dB | 0.11 | -0.14, 0.37 |
| 60-64.9 dB | -0.08 | -0.38, 0.23 |
| $\geq$65 dB | -0.13 | -0.48, 0.22 |
| School |  |  |
| <55 dB | Reference | Reference |
| 55-59.9 dB | 0.19 | -0.13, 0.51 |
| 60-64.9 dB | 0.02 | -0.39, 0.44 |
| $\geq$65 dB | -0.30 | -0.79, 0.18 |

Abbreviations: CI, confidence interval; NO_2_, nitrogen dioxide; PM, particulate matter; dB, decibel. The linear regression models were adjusted for cohort, child sex, age, ancestry, physical activity, sedentary behaviors, exposure to second-hand smoking at the 6-11 years follow-up, the family’s economic capital, maternal and paternal education, parental country of origin, maternal marital status, parity, alcohol drinking during pregnancy, and active smoking during pregnancy.

*denotes the associations which remained statistically significant after multiple testing with five effective number of tests and corrected significance level at α = 0.01.

**eFig 1. Flow chart of the study population selection**


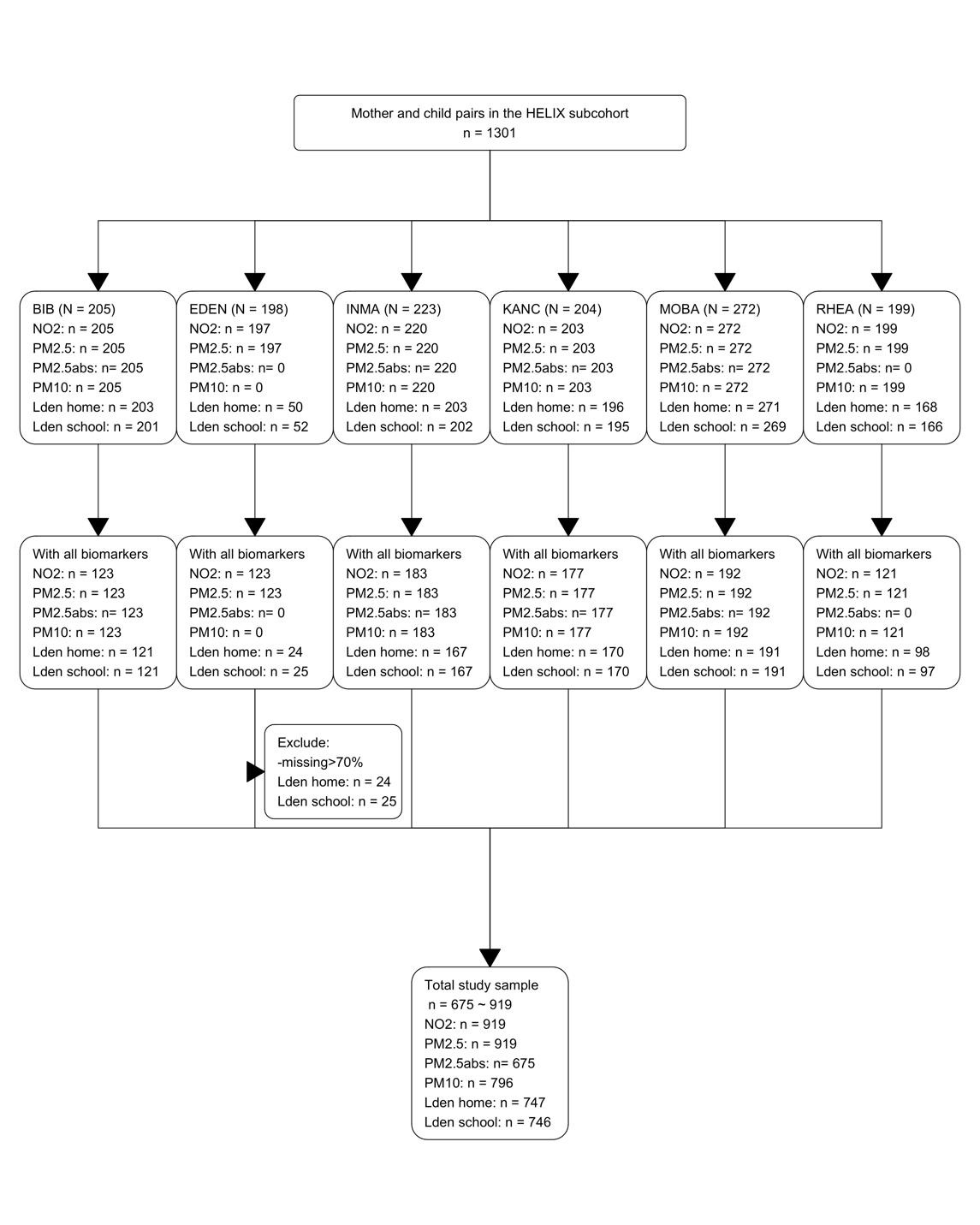


**eFig 2. Directed acyclic graph for covariates selection**
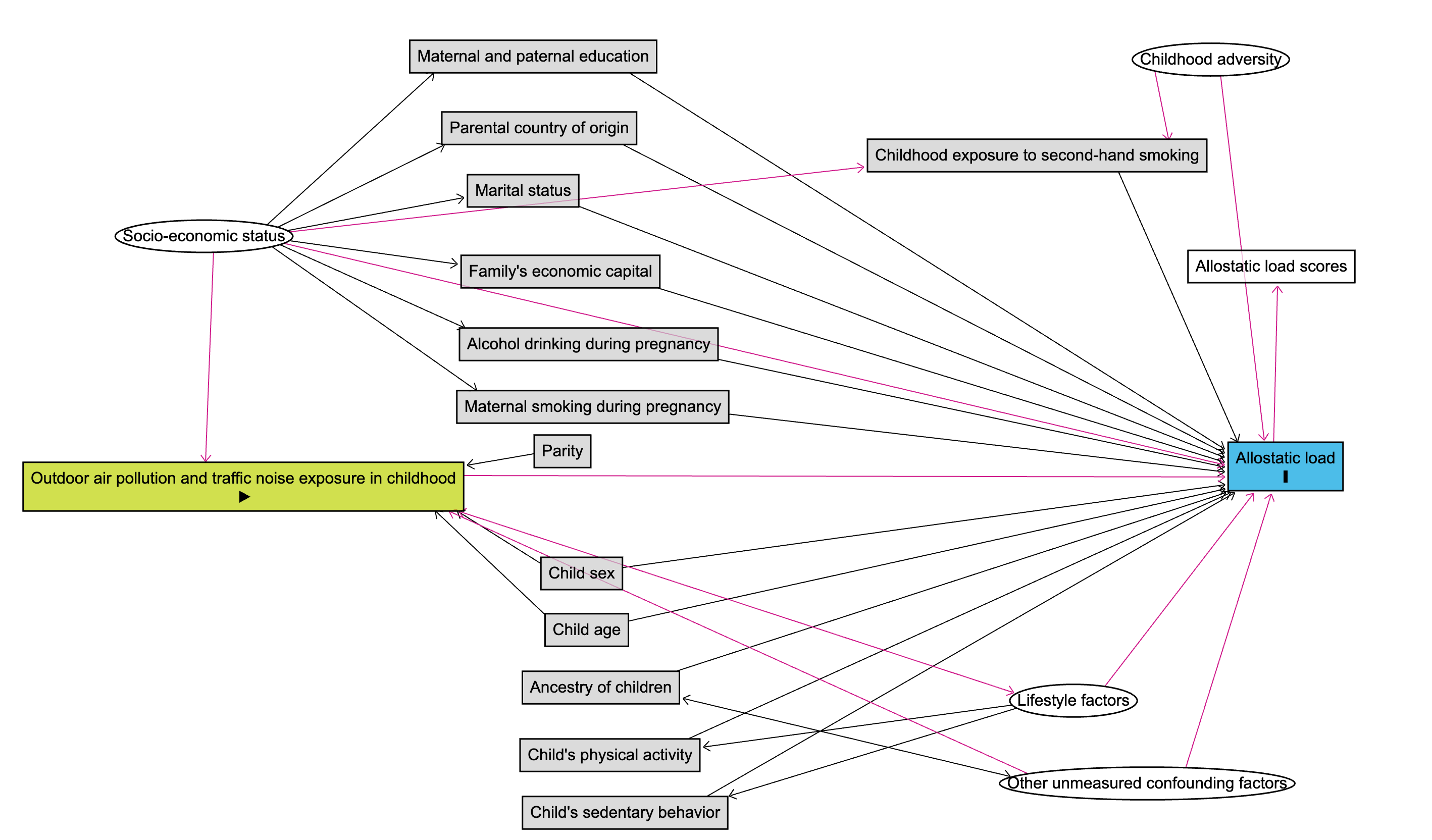


### eFig 3. Correlations of biomarkers included in allostatic load scores


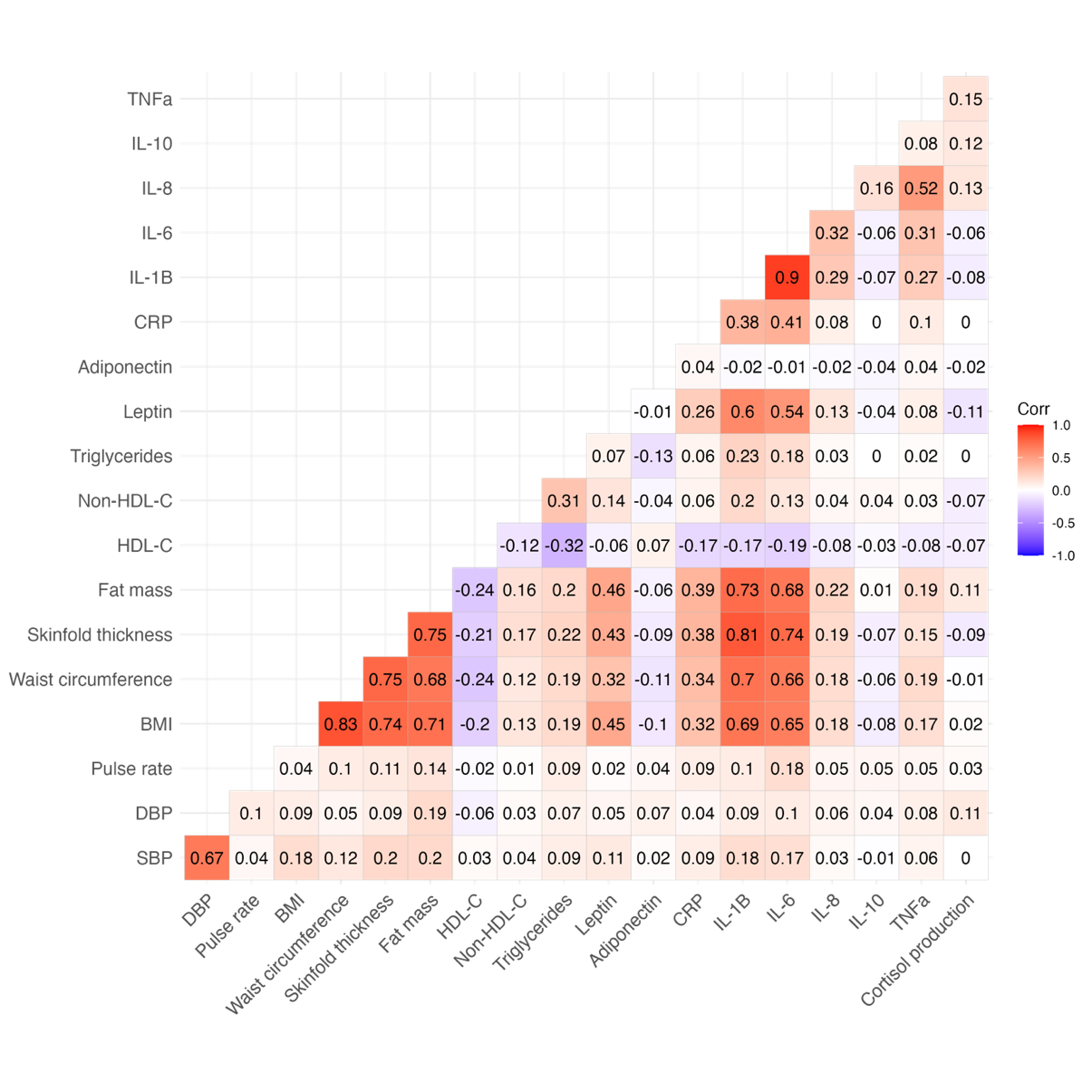


Abbreviations: SBP, systolic blood pressure; DBP, diastolic blood pressure; BMI, body mass index; HDL, high-density lipoprotein cholesterol; CRP, C-reactive protein; IL, interleukin; TNF, Tumor necrosis factor. The correlations were estimated with Pearson correlation.

**eFig 4. Correlations of allostatic load scores**


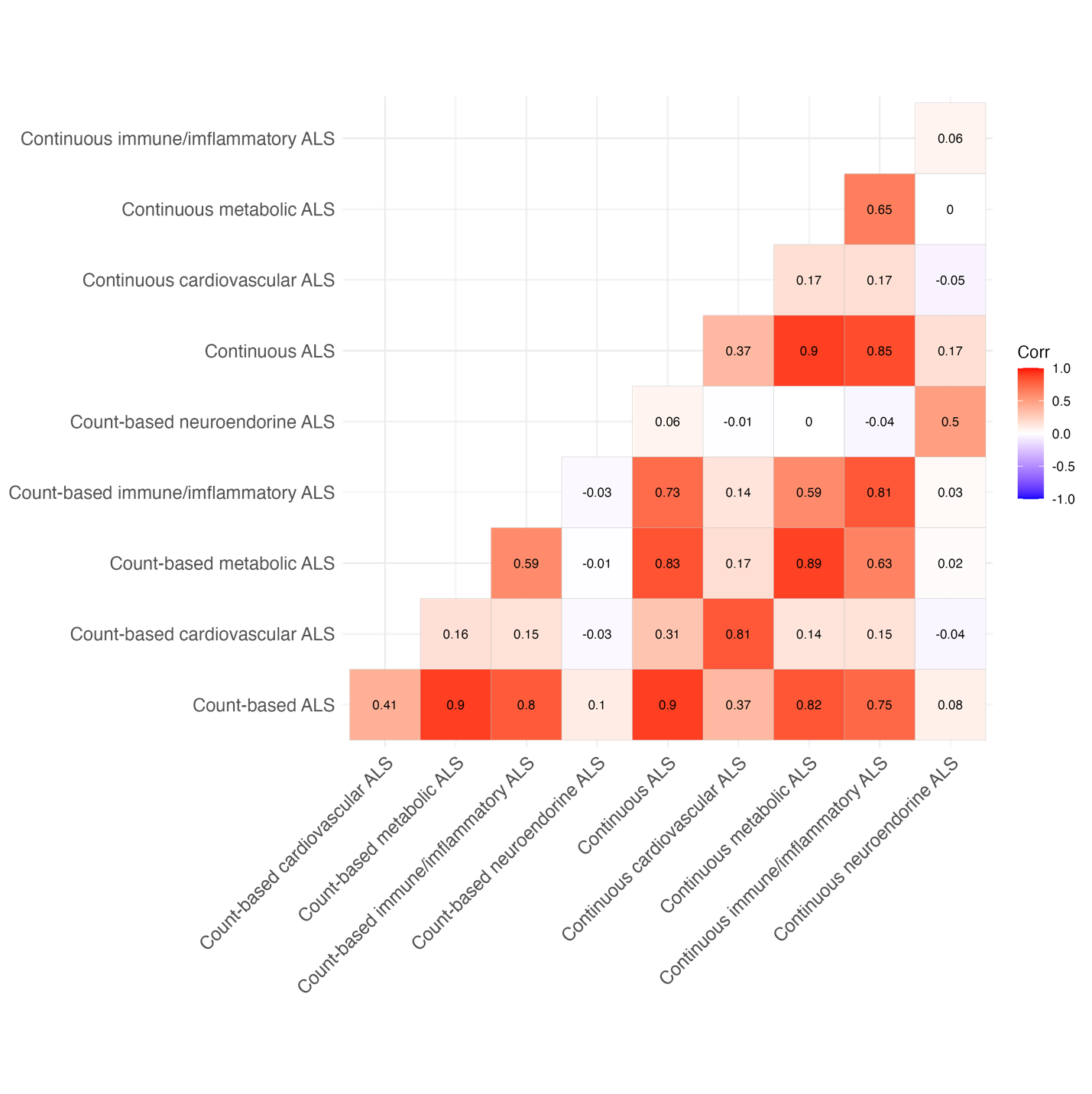


Abbreviation: ALS, allostatic load score; NO_2_, nitrogen dioxide; PM, particulate matter. The correlations were estimated with Pearson correlation. For the continuous-continuous variable pairs, Pearson correlation was used. For the continuous-categorical variable pairs, analysis of variance was used to estimate η^2^. Then correlations were calculated with square root of η^2^. For categorical-categorical pairs, Cramér's V was used.

### eFig 5. Correlations between levels of exposure to source-specific outdoor air pollutants and traffic noise and biomarkers included in allostatic load scores


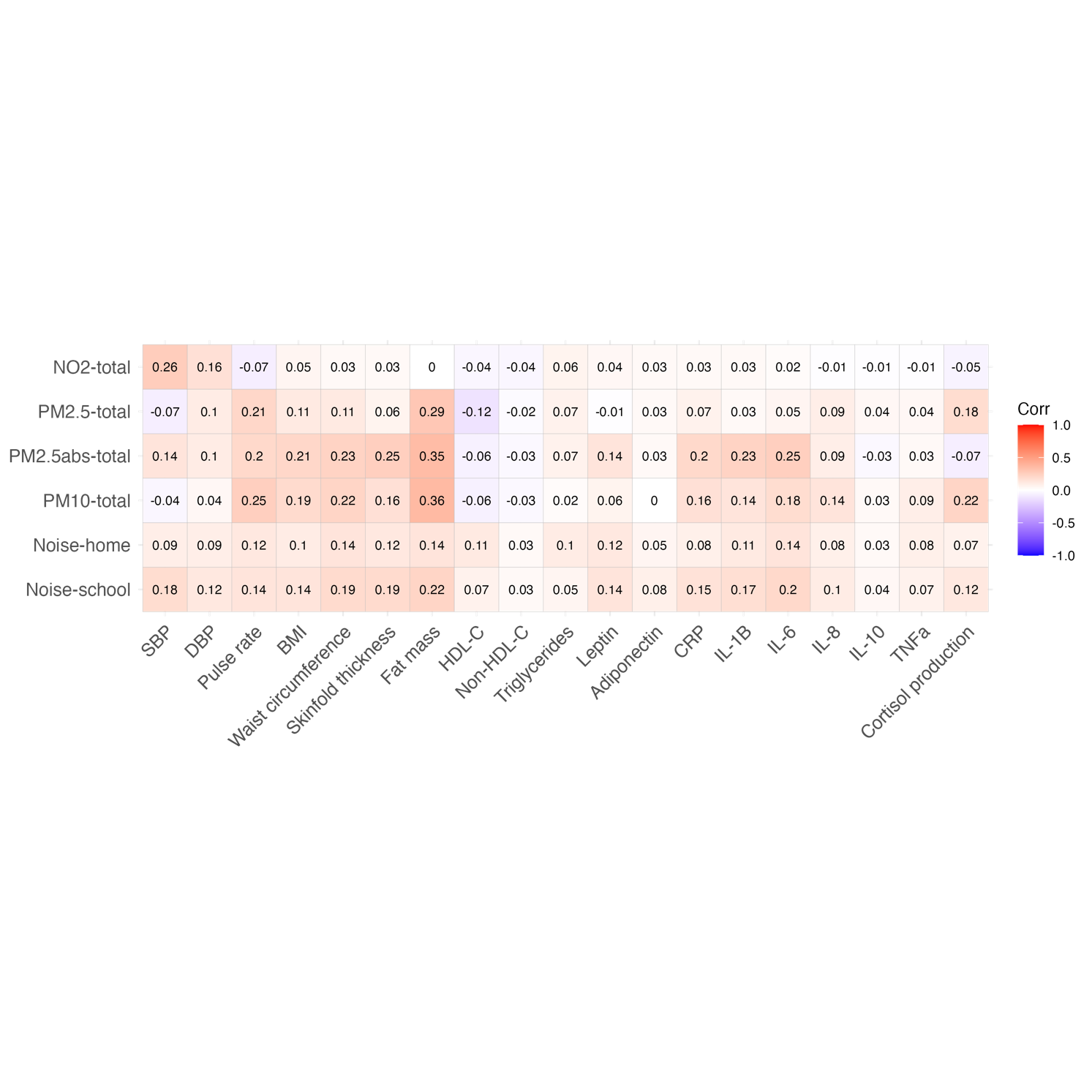


Abbreviations: CI, confidence interval; NO_2_, nitrogen dioxide; PM, particulate matter. All biomarkers were included in both count-based and continuous allostatic load scores. For the continuous-continuous variable pairs, Pearson correlation was used. For the continuous-categorical variable pairs, the correlations were calculated with square root of η^2^.

### eFig 6. Correlations of levels of exposure to source-specific outdoor air pollutants and traffic noise


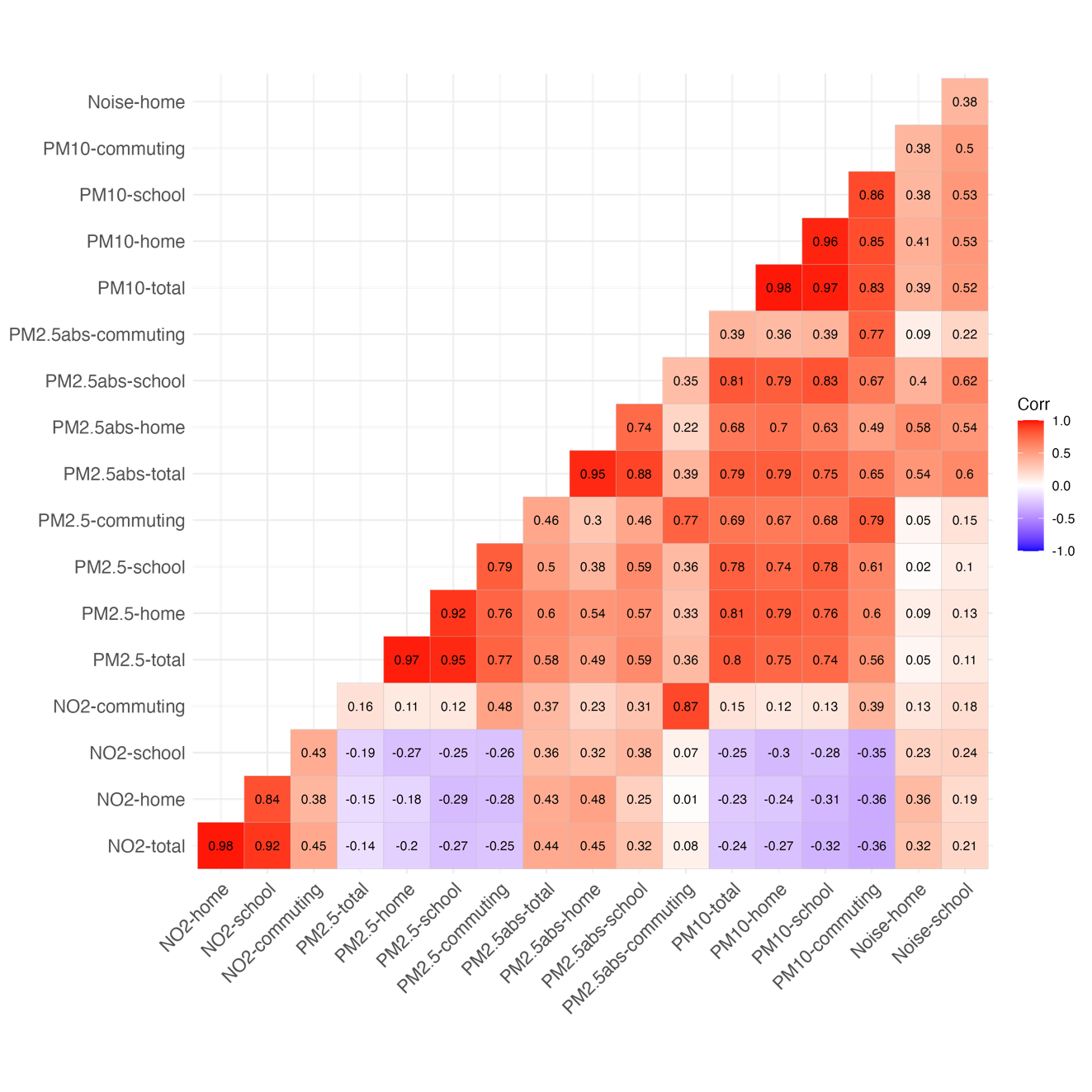


Abbreviations: CI, confidence interval; NO_2_, nitrogen dioxide; PM, particulate matter. For the continuous-continuous variable pairs, Pearson correlation was used. For the continuous-categorical variable pairs, analysis of variance was used to estimate η^2^. Then correlations were calculated with square root of η^2^. For categorical-categorical pairs, Cramér's V was used.

### eFig 7. Meta-analysis of associations between exposure to outdoor air pollutants, traffic noise and allostatic load stratified by cohort

**
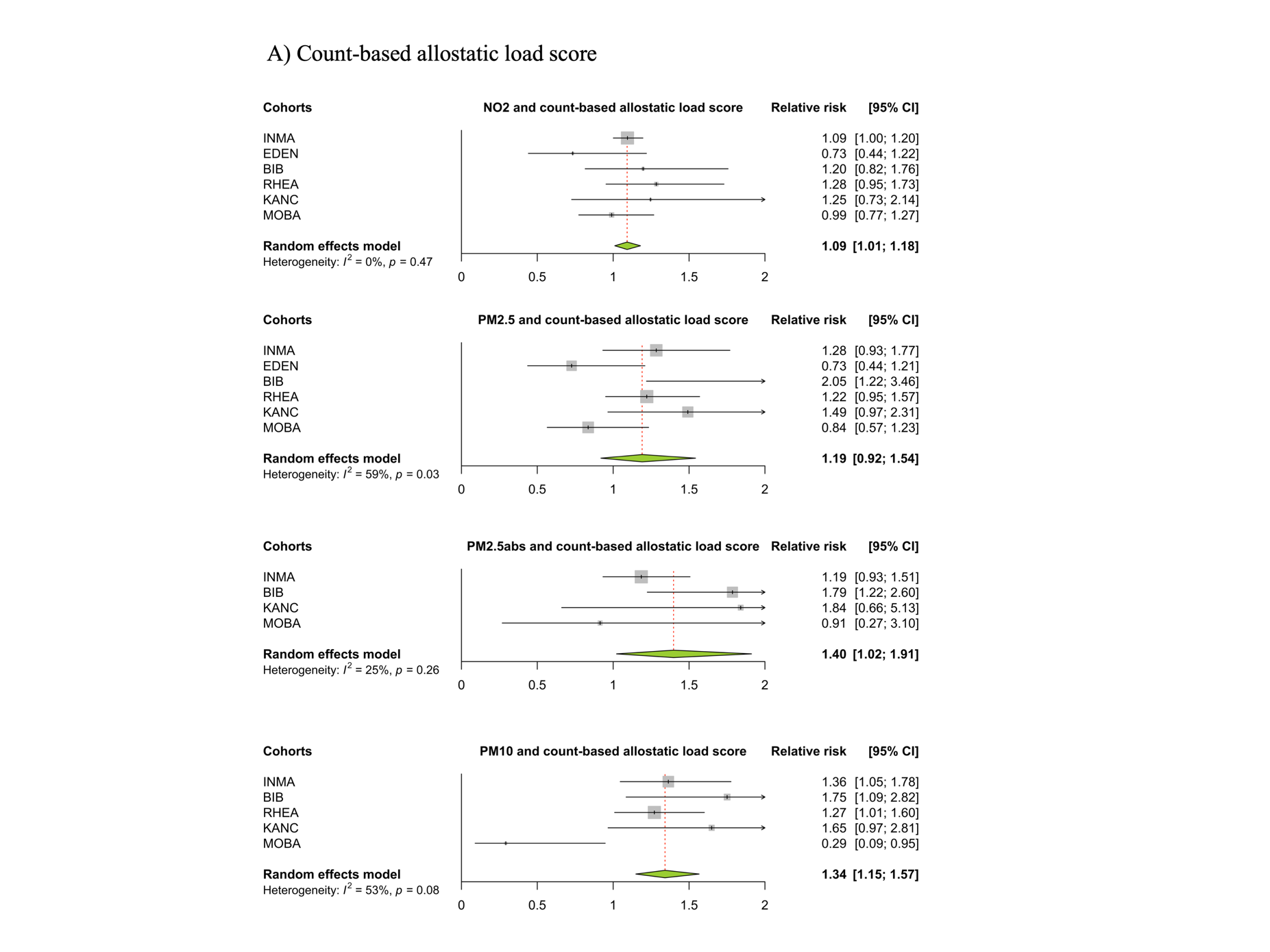
**

### eFig 7. Meta-analysis of associations between exposure to outdoor air pollutants, traffic noise and allostatic load stratified by cohort - continued

**
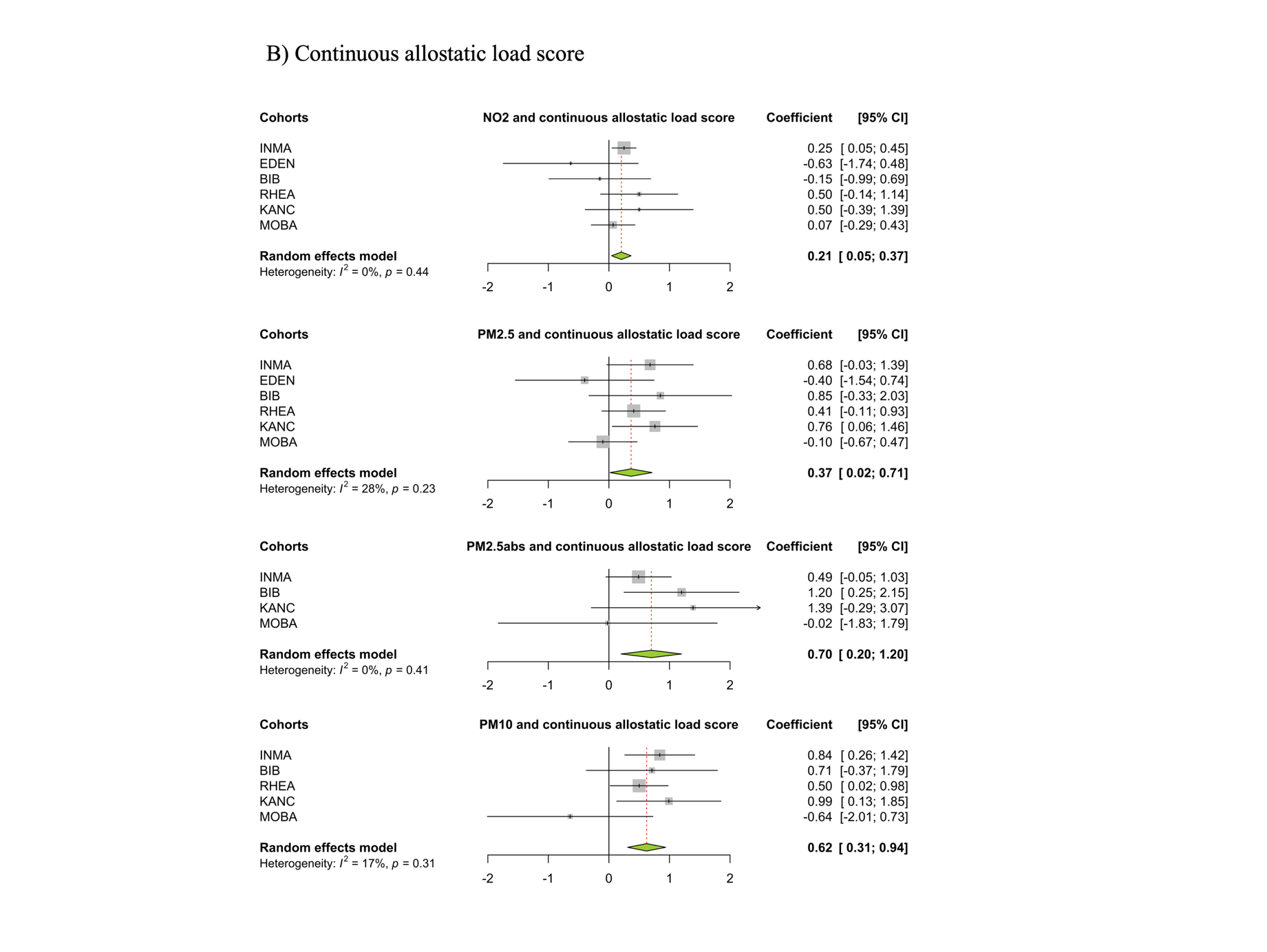
**

Abbreviations: CI, confidence interval; NO_2_, nitrogen dioxide; PM, particulate matter. The associations of outdoor air pollutants with (A) count-based allostatic load score and (B) continuous allostatic load score in each cohort, together with the pooled effect estimates using random-effect model. The models were adjusted for child sex, age, ancestry, physical activity, sedentary behaviors, exposure to second-hand smoking, the family’s economic capital, maternal and paternal education, parental country of origin, maternal marital status, parity, alcohol drinking during pregnancy, active smoking during pregnancy, child exposure to second-hand smoking at the 6-11 years follow-up.

### eFig 8. The associations of exposure to outdoor air pollutants at home, school and commuting route addresses with allostatic load

**
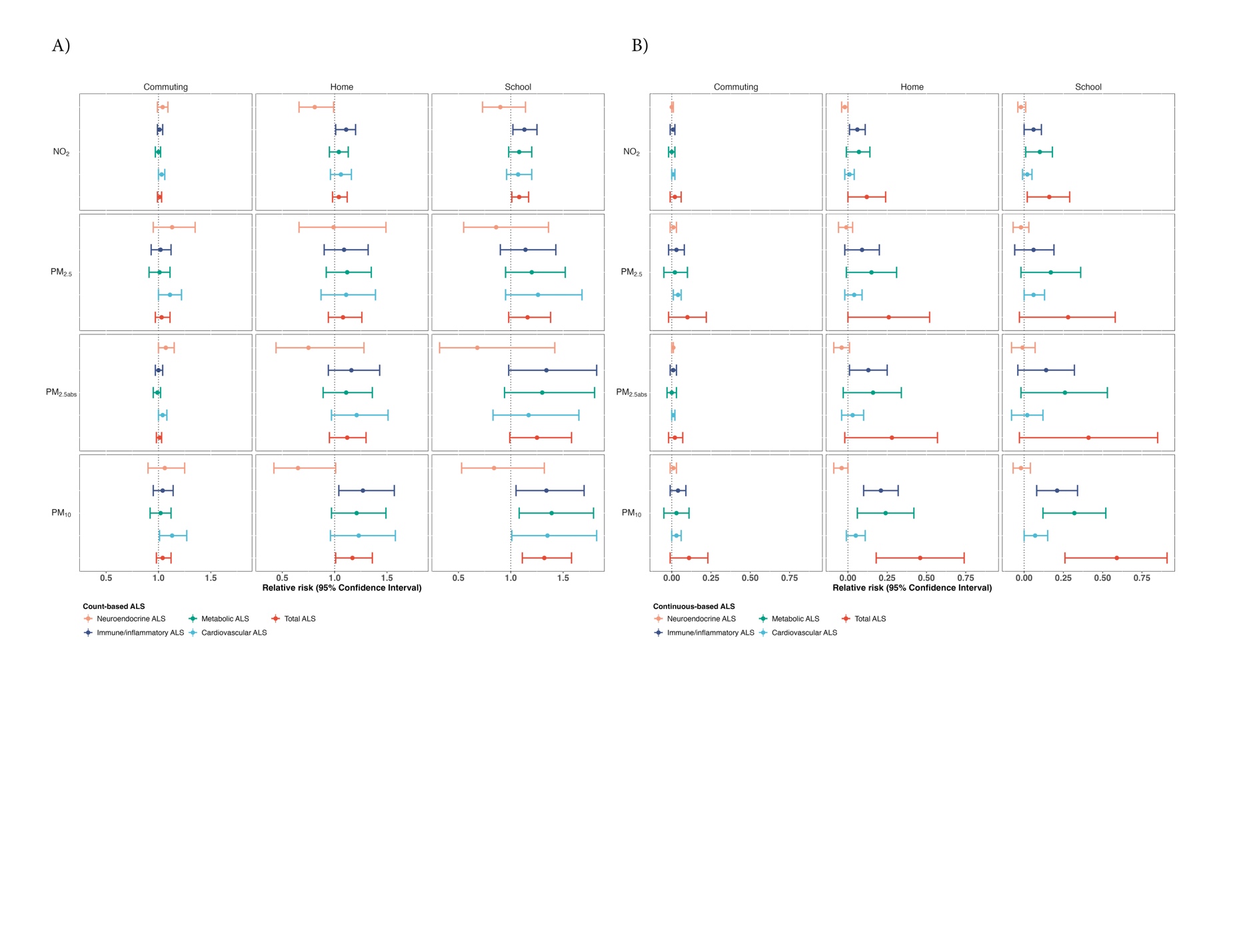
**

Abbreviations: ALS, allostatic load score; NO_2_, nitrogen dioxide; PM, particulate matter. The associations of outdoor air pollutants at home, school and commuting route addresses with (A) count-based allostatic load score and (B) continuous allostatic load score. The models were adjusted for cohort, child sex, age, ancestry, physical activity, sedentary behaviors, exposure to second-hand smoking, the family’s economic capital, maternal and paternal education, parental country of origin, maternal marital status, parity, alcohol drinking during pregnancy, active smoking during pregnancy, child exposure to second-hand smoking at the 6-11 years follow-up.

### eFig 9. The associations of exposure to outdoor air pollutants with allostatic load (excluding data from EDEN, RHEA separately and jointly)


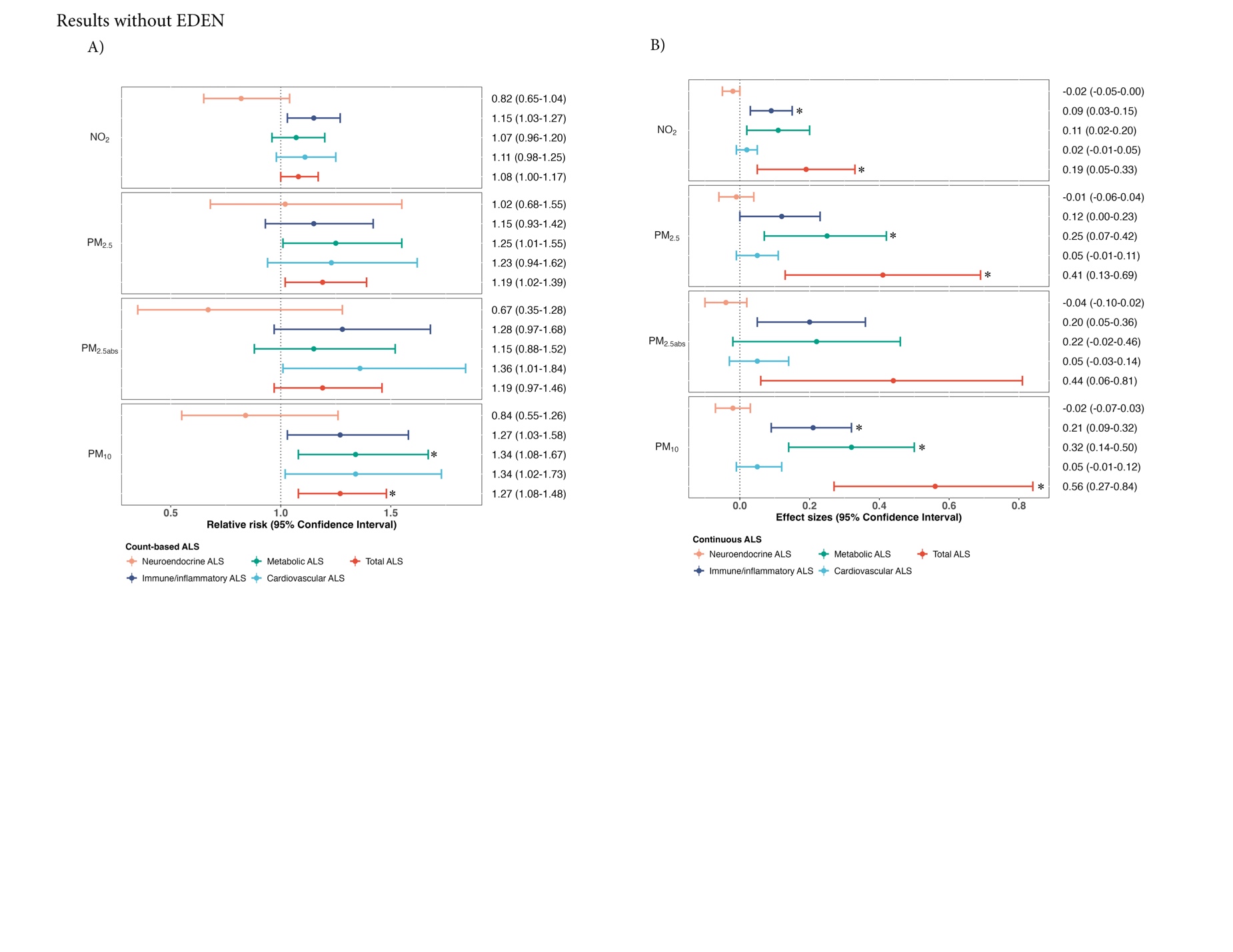


### eFig 9. The associations of exposure to outdoor air pollutants with allostatic load (excluding data from EDEN, RHEA separately and jointly) - continued


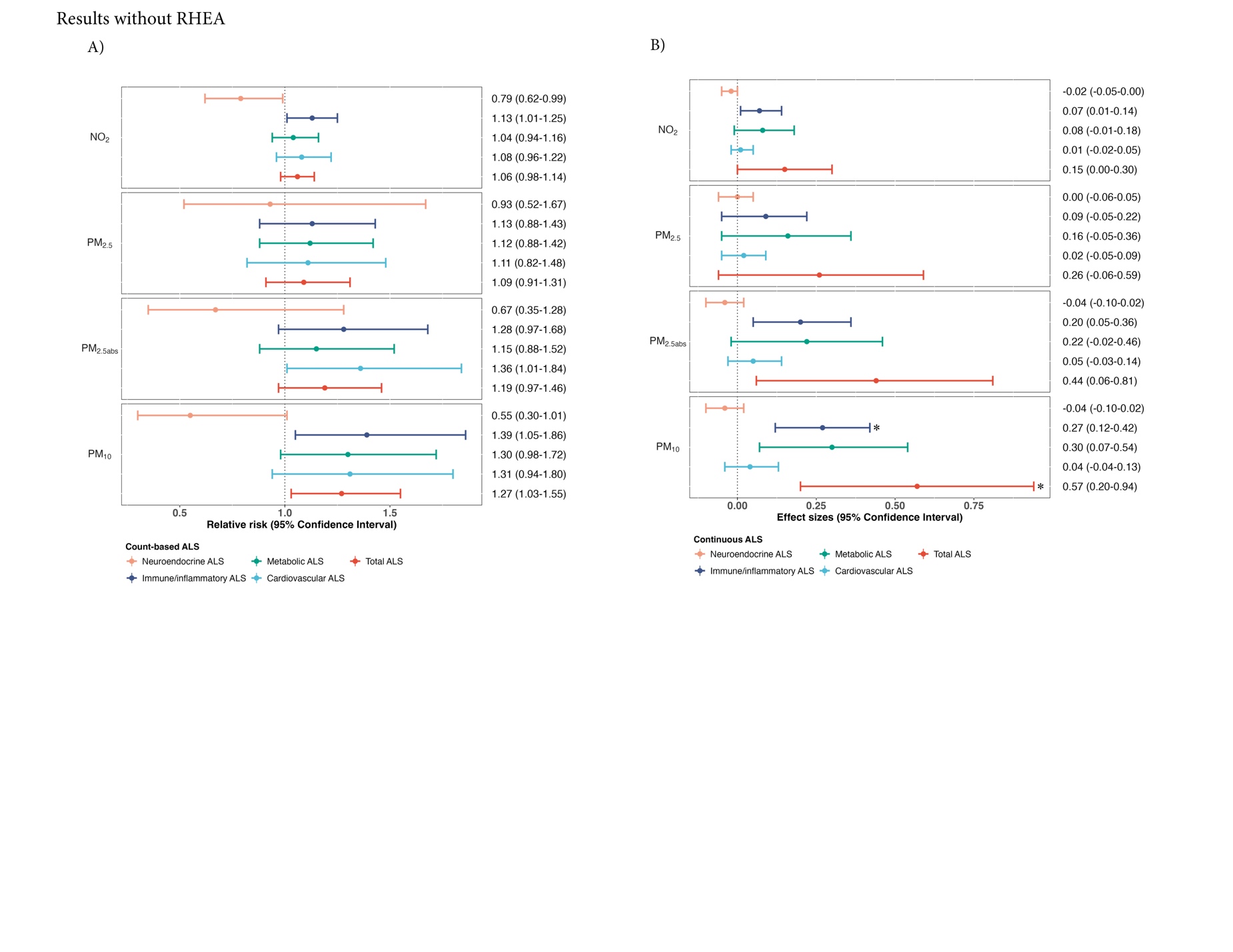


### eFig 9. The associations of exposure to outdoor air pollutants with allostatic load (excluding data from EDEN, RHEA separately and jointly) - continued


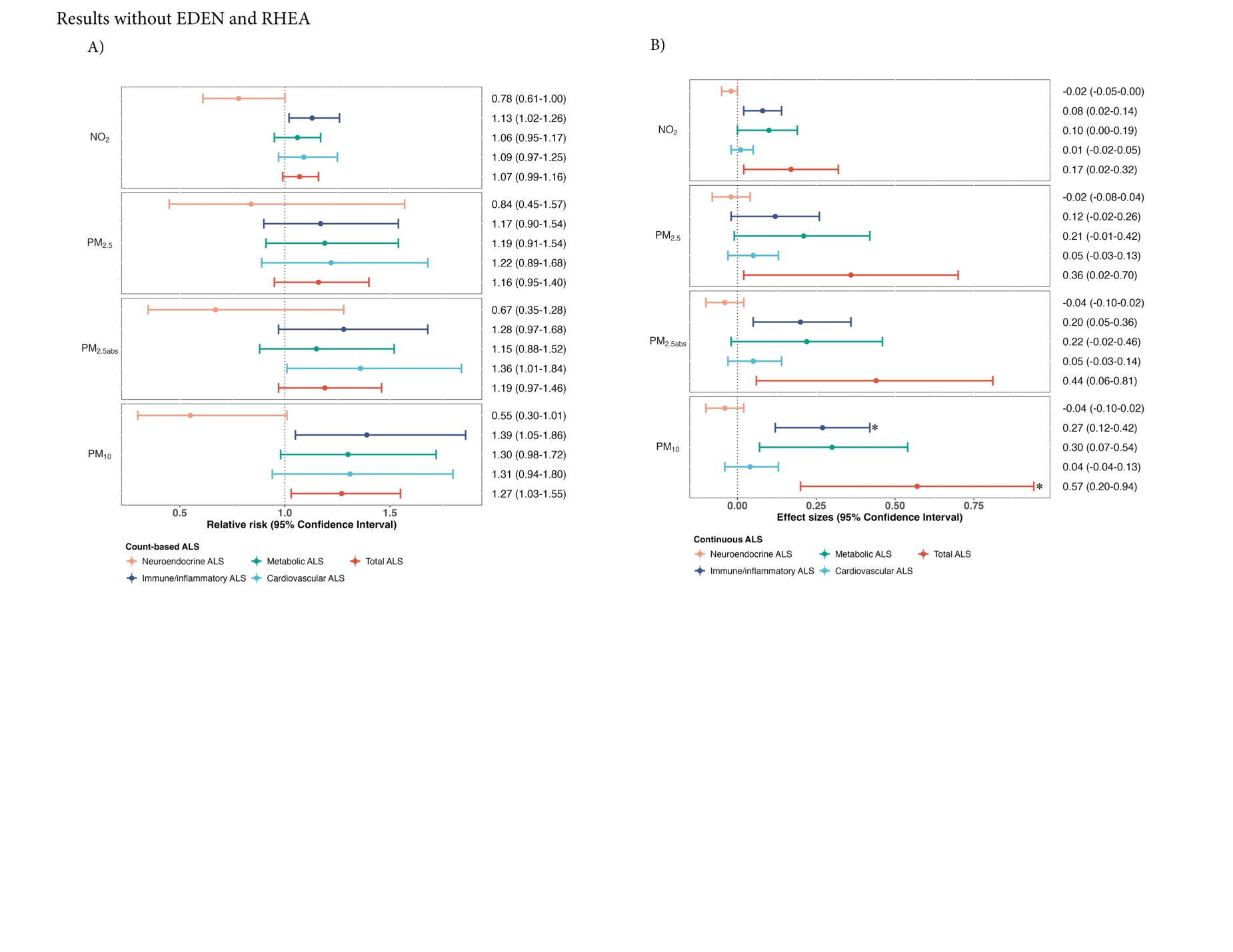


Abbreviations: ALS, allostatic load score; NO_2_, nitrogen dioxide; PM, particulate matter. The associations of outdoor air pollutants with count-based and continuous allostatic load scores in three scenarios: (A) and (B) illustrate the results without EDEN; (C) and (D) illustrate the results without RHEA; (E) and (F) illustrate the results without both EDEN and RHEA. The models were adjusted for cohort, child sex, age, ancestry, physical activity, sedentary behaviors, exposure to second-hand smoking, the Family Affluent Scale, maternal and paternal education, parental country of origin, maternal marital status, parity, alcohol drinking during pregnancy, active smoking during pregnancy, child exposure to second-hand smoking at the 6-11 years follow-up.

* denotes statistical significant after multiple testing correction (p-value 0.01).
